# Deep and high-resolution three-dimensional tracking of single particles using nonlinear and multiplexed illumination

**DOI:** 10.1038/ncomms8874

**Published:** 2015-07-29

**Authors:** Evan P. Perillo, Yen-Liang Liu, Khang Huynh, Cong Liu, Chao-Kai Chou, Mien-Chie Hung, Hsin-Chih Yeh, Andrew K. Dunn

**Affiliations:** 1 Department of Biomedical Engineering, The University of Texas at Austin, 107 W Dean Keeton Street, C0800, Austin, Texas 78712, USA; 2 Department of Molecular and Cellular Oncology, The University of Texas MD Anderson Cancer Center, 1515 Holocombe, Boulevard, Unit 108, Houston, Texas 77030-4009, USA; 3 Center for Molecular Medicine and Graduate Institute of Cancer Biology, China Medical University, No. 91 Hsueh-Shih Road, Taichung 40402, Taiwan

## Abstract

Molecular trafficking within cells, tissues and engineered three-dimensional multicellular models is critical to the understanding of the development and treatment of various diseases including cancer. However, current tracking methods are either confined to two dimensions or limited to an interrogation depth of ∼15 μm. Here we present a three-dimensional tracking method capable of quantifying rapid molecular transport dynamics in highly scattering environments at depths up to 200 μm. The system has a response time of 1 ms with a temporal resolution down to 50 μs in high signal-to-noise conditions, and a spatial localization precision as good as 35 nm. Built on spatiotemporally multiplexed two-photon excitation, this approach requires only one detector for three-dimensional particle tracking and allows for two-photon, multicolour imaging. Here we demonstrate three-dimensional tracking of epidermal growth factor receptor complexes at a depth of ∼100 μm in tumour spheroids.

## Abstract (web summary)

Existing single-particle tracking techniques are limited in terms of penetration depth, tracking range or temporal resolution. Here, Perillo *et al*. demonstrate three-dimensional particle tracking up to 200-μm depth, with 35-nm spatial localization and 50-μs resolution using multiplexed two-photon excitation.

## Graphical abstract

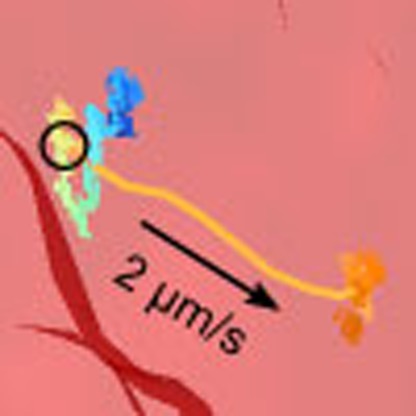

Single-particle tracking (SPT) has enabled the direct observation of dynamic behaviours of particles (here a particle can be a single biomolecule, a molecular complex, a vesicle, a lipid granule or a viral capsid) inside complex biological systems^1^,^2^,^3^,^4^, with particle localization precision better than the diffraction limit of light^5^,^6^. By trajectory analysis, SPT has provided insight into motor protein kinetics^7^,^8^, cellular membrane dynamics^9^,^10^,^11^, mRNA transport^12^,^13^ and virus internalization processes^14^,^15^. As the basis of passive microrheology, SPT has also shed light on the local environments of tracked particles through the observation of changes in particles' random movements^16^,^17^.

Whereas SPT is becoming a powerful research tool, all current techniques suffer from one or more of the following problems: shallow penetration depth (arising from the use of one-photon excitation^18^,^19^,^20^), limited *z* tracking range (for example, total internal reflection fluorescence microscopy), poor temporal resolution (for example, frame-by-frame analysis in camera-based methods^21^,^22^) and low information content (for example, no information on the fluorescence lifetime^23^). As two-photon (2P) microscopy has become a standard method for deep tissue imaging^24^, a few reports demonstrated three-dimensional (3D) tracking based on 2P excitation. One of the earliest demonstrations of 2P tracking used an orbital scanning motion of the focused laser beam to track particles using a single detector^5^,^25^,^26^, but was limited to a temporal resolution of 20–32 ms due to mechanical scanning and signal demodulation. More recently, 3D tracking of gold nanorods with 2P excitation was demonstrated by exciting multiple foci and detecting fluorescence with an Electron Multiplying Charge Coupled Device (EMCCD)^22^, but the 3D temporal resolution was limited to ∼0.5 s. Moreover, the use of a camera in multifocal 2P laser scanning microscopy limits the working depth of SPT in scattering samples^27^. Although SPT with superior temporal resolution (bounded mainly by the emission rate of the fluorescent label) and simultaneous fluorescence lifetime measurements have been achieved using confocal setups with 3–5 single-element/photon-counting detectors (photomultiplier tubes (PMTs) or avalanche photodiodes) for spatial filtering^18^,^19^,^20^, these methods not only have limited working depth (not using 2P excitation for tracking) but also suffer from loss of signals due to the non-overlapping excitation and collection efficiency peaks in spatial filtering (Supplementary Fig. 3)^28^. Other confocal-based microscopes have been developed using only two detectors, which are capable of simultaneous spectroscopy measurements^29^; however, they still suffer from poor penetration depths in scattering samples such as tissues and multicellular structures, as well as low collection efficiency. Currently there is no single solution to all of the above issues.

To address this challenge, we have developed a 2P 3D SPT method capable of tracking particles at depths up to 200 μm in scattering samples with 22/90 [*xy*/*z*]-nm spatial localization precision and 1 ms response time. With bright fluorophores, we can achieve a temporal resolution down to 50 μs. At shallow depths, the localization precision can be as good as 35 nm in all 3D. The approach is based on passive pulse splitters used for nonlinear microscopy^30^ to achieve spatiotemporally multiplexed 2P excitation and temporally demultiplexed detection^31^ to discern the 3D position of the particle. The z-tracking range is up to ±50 μm (limited by the objective z-piezo stage) and the method enables simultaneous fluorescence lifetime measurements on the tracked particles. Like some more recent techniques, this tracking method allows coupling trajectory data with traditional imaging to discern the local environment^18^,^32^. However, a major advantage of this method over confocal approaches is that it requires only one detector for SPT and is compatible with multicolour 2P microscopy. We describe our approach and demonstrate its capabilities by tracking single fluorescent beads in aqueous solutions that include scattering, as well as tracking prescribed motions in these controlled environments. We then demonstrate tracking of epidermal growth factor receptor (EGFR) complexes tagged with fluorescent beads in tumour spheroids, demonstrating deep 3D SPT in multicellular models. We have coined this technique tracking single particles using nonlinear and multiplexed illumination (TSUNAMI).

## Results

### Spatiotemporal multiplexer design

**Figure 1:**
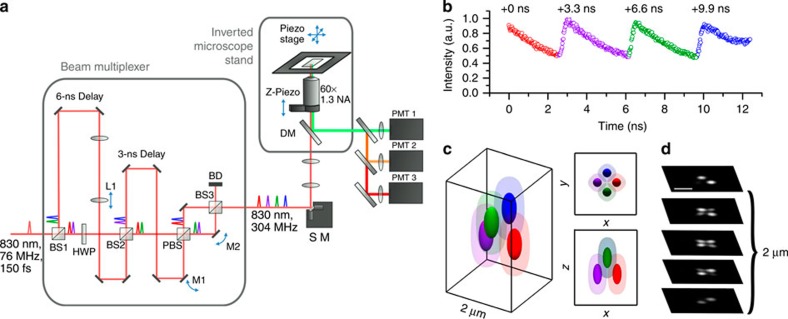
3D tracking instrumentation. (**a**) Schematic of the two-photon 3D tracking microscope. Spatiotemporal multiplexing is enabled through an optical system which utilizes two beam splitters (BS1 and BS2) to generate four beams, which can be quasi-independently controlled via mirrors (M1 and M2). Physical delay lines provide temporal separation. In this case, 6.6 ns (2 m) and 3.3 ns (1 m) path length delay lines create four beams with a period of 3.3 ns corresponding to an even division of the fundamental 13-ns period generated by the laser source (Mira 900, Coherent). Tracking actuation is performed using scanning mirrors (SM) and an objective focusing stage (z-piezo). (**b**) Photon-counting histogram of a particle centred in the middle of the four excitation focus demonstrating temporal offsets and power balance between the independent excitation beams. (**c**) An idealized image space projection of the tetrahedral PSF. (**d**) Experimental laser scanning image of a single φ100-nm fluorescent bead with simultaneous four beam excitation. Scale bar, 1 μm. HWP, half-wave plate; PBS, polarizing beam splitter; DM, dichroic mirror; BD, beam dump.

In the spatiotemporal multiplexed scheme, laser pulses emitted with a 13-ns period from a Ti:sapphire oscillator are separated into four beams, which are delayed by 3.3 ns each and focused through a high numerical aperture objective at slightly offset *xyz* positions. The four resulting 2P excitation volumes are arranged into a barely overlapped, tetrahedral geometry (Fig. 1c), to generate selective excitation equivalent to the spatial filtering condition in the previous four-detector confocal tracking set-up^19^, with each 2P excitation volume receiving laser pulses at a different time delay. For a fluorescent particle residing somewhere inside the excitation tetrahedron, its 2P emission is collected by a PMT (PMT1 in Fig. 1a). By time-correlated single-photon-counting (TCSPC) detection, each detected photon is assigned to a specific time gate (G_1_–G_4_, here assuming the decay time is ∼4 ns or less) in the fluorescence decay histogram (Fig. 1b), and therefore attributed to an individual excitation volume. For a particle sitting at the centre of the tetrahedron, the resulting photon counts are approximately equal in all four time gates. An offset of the particle from the tetrahedron centre can be estimated from the normalized photon count differences in the four time gates (that is, error signals *E*_*x*_, *E*_*y*_ and *E*_*z*_ in Supplementary Methods). Once the particle position offset is determined, a closed feedback loop then steers galvanometer mirrors and the objective z-piezo stage to lock the tracking beams on the particle. A particle's 3D trajectory is therefore determined directly from the controller output sent to the galvanometer and piezo actuators.

Spatiotemporal multiplexing has previously been explored for diffusion measurements^33^ and tracking^34^, but these methods rely on a picosecond pulsed laser for one-photon excitation and therefore are not suitable for use in multicellular models or tissues. Furthermore, only one photomultiplier tube (PMT1 in Fig. 1) is needed for SPT in our method, whereas 3–5 detectors are needed in confocal tracking set-ups^18^,^19^,^20^. In addition, the fluorescence lifetime of the tracked particle can be determined from the time-resolved photon data so long as the emitter lifetime is shorter than the gate width (<3.3 ns; Supplementary Fig. 12) (ref. 26). As the laser beam is steered by active feedback to lock on the tracked particle, a large tracking range is achieved (±50 μm in *z* direction and ±100 μm in *xy* direction) with minimal perturbation to the samples (whereas some confocal setups require the sample to be moved in order for SPT^18^,^19^).

### Calibration and localization precision characterization

**Figure 2:**
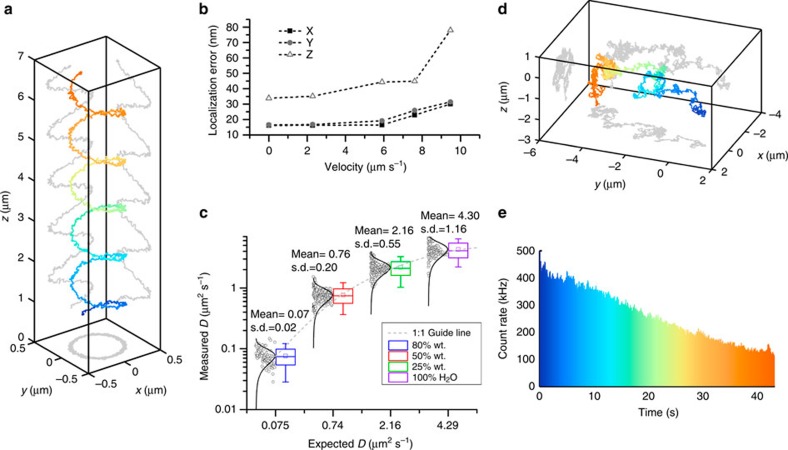
Characterization of directed motion and free diffusion. (**a**) Example helical trajectory of a φ100-nm fluorescent bead moved through a known path using an independent 3D piezo stage (P-733K130, PI). The path duration was 7 s with an average velocity of 2.1 μm s^−1^. The tracking system reproduced the true trajectory accurately with 16.5 nm uncertainty in *x* and *y* and 33.3 nm in *z*. Rainbow coloration corresponds to time with blue representing the beginning of the trajectory. (**b**) Several helical trajectories were performed with varying mean velocities. The particle localization uncertainty increases with increasing velocities up to 10 μm s^−1^, where the particle speed is too fast for the controller to target lock. (**c**) Box plot with histograms of measured versus theoretical diffusion coefficients, *D*, for 80% wt. glycerol, 50% wt. glycerol, 20% wt. glycerol and 100% water, respectively. The measured central tendency of the diffusion coefficients were found to agree with the Stokes-Einstein equation for wide range of values (0.07–4.3 μm^2^ s^−1^). The agreement with theory can been seen by how closely the data follow the 1:1 guide line (plotted on semilog scale). (**d**) An example trajectory of a φ100-nm fluorescence bead in an 80% wt. glycerol solution, free diffusion was observed over 43 s. (**e**) Particle photon count rate versus time, a monotonically decreasing count rate indicates that a single bead, or aggregate, is stably locked in the field of view for the entire duration of the trajectory with no other particles entering or leaving the field of view.

To validate our TSUNAMI microscope, we first tracked fluorescent beads (φ100 nm, decay time ∼4.5 ns) in aqueous solution (Fig. 2) and in 9% gelatin gel with 1% intralipid (a highly scattering environment; Supplementary Fig. 7). By following prescribed motions in these controlled environments^5^,^35^, we successfully characterized the localization precision, tracking speed limits, temporal resolution and tracking depth of our system.

From optical modelling, the optimal lateral and vertical separation distances between the 2P excitation volumes were estimated to be 500 and 1,000 nm, respectively (Supplementary Fig. 3)^28^. The alignment of the four excitation beams was verified by volumetric scanning with a fixed fluorescent bead (Fig. 1d). To determine particle localization uncertainty and maximum speed that our system can follow, we tracked a fixed fluorescent bead (φ100 nm, F-8803, Life Technologies) loaded on an independent *xyz* piezo stage (P-733K130, PI)^5^,^35^. The independent stage was programmed to move in a helical pattern (Fig. 2a). At an average speed of 2 μm s^−1^, the estimated tracking errors (root mean squared) were 16.2 nm in *x*, 16.7 nm in *y* and 35.1 nm in *z* (Supplementary Fig. 6). The localization precision stayed below 45 nm when the particle speed was <8 μm s^−1^ (Fig. 2b). It should be noted that the fastest molecular motor known today^36^, FtsK, travels at ∼7 μm s^−1^. Other than prescribed motions, we also tracked freely diffusing nanoparticles (φ100 nm, F-8803, Life Technologies) at various diffusion rates. Diffusion coefficients were estimated from fitting the mean-square displacement and compared with the values predicted from the Stokes-Einstein equation. Excellent agreements were seen in a wide range of diffusion coefficients (0.07–4.3 μm^2^ s^−1^ in Fig. 2c; Supplementary Fig. 5). Whereas a previous 3D SPT report has successfully tracked particles diffusing at 20 μm^2^ s^−1^ (ref. 18), we note that diffusion coefficient of a free receptor complex on the cell membrane is roughly on the order of 0.02 μm^2^ s^−1^ (ref. 37), and the fast diffusion coefficient of proteins in the cytosol is on the order of 5 μm^2^ s^−1^ (ref. 38). Our system thus has no problem to probe rapid molecular transport dynamics inside cells.

### Temporal resolution characterization

Temporal resolution of our tracking system is defined as how fast the particle position is discerned in 3D space with reasonable localization accuracy. Although our control loop period is 1–5 ms, we emphasize that our temporal resolution can be significantly better than 1 ms by outputting the individual photon event data (Time Tag) from the TCSPC board (while the control loop period remains at 1–5 ms). In offline analysis, trajectories can be resampled with temporal resolution down to 50 μs if the particle has a sufficiently high brightness (Supplementary Fig. 11). In this condition, the trajectory is plotted from a combination of the original control loop rate voltage outputs and *n*-samples of higher time resolution localizations relative to the current beam position. Localizations are performed using rebinned histograms with photons arriving only within the super-sampled time period.

### Tracking depth characterization

To mimic 3D tracking in a turbid tissue sample, φ40 nm fluorescent beads were fixed within a 9% gelatin gel with 1% intralipid (Supplementary Fig. 7). When tracking beads undergoing prescribed motion at ∼10-μm depth, localization uncertainty stayed the same in *xy* direction but slightly increased in *z* direction (60 nm). Localization uncertainty further increased to ∼89 nm at the depths of 100 through 200 μm, while localization uncertainty stayed below 22 nm in *xy* direction at both depths. This reduction in *z* localization precision may be due to an elongated molecular detection function (Supplementary Fig. 4) that occurs when light is focused through scattering samples, which may blur the beams along the *z* dimension^39^ and lower the optical contrast signal required to lock onto the target. Despite this slight reduction in *z* tracking accuracy at depth, TSUNAMI is capable of maintaining better than 100-nm axial localization through 200 μm of a scattering sample.

### 2P-3D-SPT in monolayer cultures and cancer spheroids

**Figure 3:**
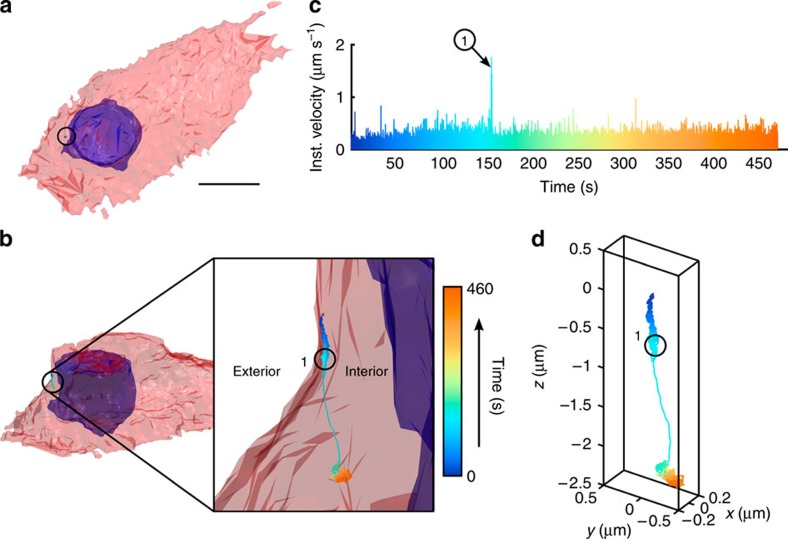
3D single-particle tracking in monolayer cell cultures. The capability to track singly labelled EGF molecules is demonstrated in a live-cell environment. (**a**) 3D isocontour model of the cell structure with staining for plasma membrane (red) and nuclei (blue). Scale bar, 10 μm. (**b**) Cell isocontour model plotted with trajectory overlaid (inset: zoomed view of the particle trajectory, a single fluorescent-labelled EGF molecule was tracked for a duration of 460 s with rainbow coloration corresponding to time, where blue marks the beginning). (**c**) Instantaneous velocity graph with corresponding colour scheme. For the first 150 s, the particle undergoes slow directed diffusion (mean velocity ∼0.4 μm s^−1^) along the exterior of the cell. A ramp in velocity is observed followed by a period of high average velocity (start denoted as point 1), ∼2 μm s^−1^, and unidirectional transport of 1 μm. This behaviour indicates some form of internalization into the cell or transport within the cell. (**d**) Trajectory plotted with no cell contour overlay.

**Figure 4:**
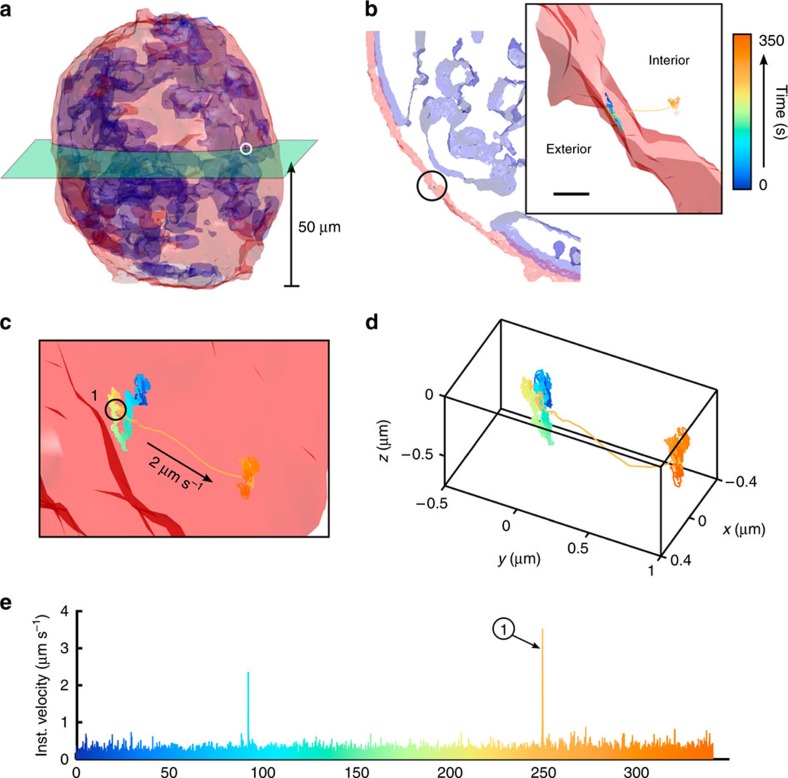
Deep single-particle tracking of EGFR in spheroid model. Demonstration of deep 3D SPT in a spheroid model. (**a**) 3D isocontour of a φ100-μm spheroid taken with 2P laser scanning microscopy staining for the plasma membrane (red) and nuceli (blue). The highlighted slice denotes the *z* plane (at 50-μm depth) where the trajectory was measured, with the white circle marking the location of the trajectory on the spheroid. (**b**) Isocontour model of the ±5-μm slice taken 50-μm deep within the spheroid. Plasma membrane and nuclei are overlaid with the trajectory (black circle), (inset: zoomed view of the trajectory). (**c**) Zoomed view of the trajectory. The trajectory begins inside the cell with slow displacement (mean velocity ∼0.17 μm s^−1^) for 250 s. Point 1(black circle) is where velocity increases to 2 μm s^−1^and is sustained for 0.5 s in a unidirectional manner. (**d**) Trajectory plotted without cell overlay, the total transport length within the high velocity region is 1 μm. (**e**) Instantaneous velocity plot over the duration of the trajectory. Scale bar, 600 nm.

Here we used the endocytosis and subcellular trafficking of EGFR complexes in A431 monolayer culture and tumour spheroids as a model system for instrument validation. We tracked single-nanoparticle (φ40 nm, F8770, Life Technologies)-bound EGFR complexes (see Supplementary Online Methods) in monolayer cell cultures (Fig. 3a) and ∼φ100-μm spheroids (Fig. 4a) for periods up to 10 min. Before SPT, 2P fluorescence images were taken of the surrounding cellular environment. Staining of the plasma membrane and nucleus allows co-registration of the particle trajectory with cellular landmarks. In post processing, the trajectory and cellular images are co-registered (Fig. 3b) to visualize EGFR entry pathways.

We found that out of 100 trajectories ∼80% of EGFRs had been internalized into the cells within ∼6 min. We notice transport modes similar to those described in prior work^40^, (Fig. 3d). The average velocity (2 μm s^−1^) and total transport length (1–2 μm) during internalization are in good agreement with values previously reported.

For spheroid models, we measured EGFR entry pathways at a variety of depths from 20 to 100 μm past the coverslip (Fig. 4c; Supplementary Figs 13 and 14) and found good agreement in terms of the speed and transport length during internalization (Fig. 4d). Although the required power to obtain clean spheroid images expectedly increased with imaging depth, the total signal count rate (500–800 kHz) and signal to noise remained well within a threshold required for target locking. EGFR trajectories were easily measured at a depth of 100 μm for up to 10 min with minimal photobleaching. To evaluate whether the trajectories are representative of biological events or system artifacts, we measured trajectories under a control environment with an endocytosis inhibitor, sodium azide and low temperature (<20 °C) (Supplementary Fig. 15). Of the 30 inhibited trajectories, we observed no high-speed transport modes and only external membrane-bound slow diffusion with an average velocity of ∼0.2 μm s^−1^. The average tracking duration was 500 s.

Clearly this technique is capable of measuring biologically relevant activity in the high-background environment of monolayer and spheroid models with instantaneous transport speeds up to 7 μm s^−1^. We emphasize that TSUNAMI is capable of measuring EGFR translocation pathways at depths 10 × what was previously possible through highly scattering, cell dense samples. In addition, the multicolour, multiresolution 2P fluorescence imaging functionality is inherently integrated to allow for co-registration of deep trajectories to local cellular/tissue environment in 3D.

## Discussion

Whereas a few reports demonstrated 3D SPT in monolayer cell cultures using 2P excitation^5^,^22^, to the best of our knowledge high-resolution subsurface particle tracking in 3D has never been demonstrated in highly scattering multicellular environments. One recent report demonstrates deep 3D SPT light sheet microscopy using astigmatism^41^; however, the capability is limited by the use of an EMCCD (16-ms temporal resolution), and would prove difficult to implement multicolour tracking or simultaneous lifetime measurement schemes. Furthermore, we expect an astigmatic point spread function (PSF) would have significantly decreased localization precision when tracking at depth (greater than 10 μm)^21^.

Other than penetration depth, 2P excitation allows us to excite multiple fluorophores simultaneously, which greatly facilitates multicolour detection (Fig. 3)^42^. The two factors that determine the fundamental limit of our temporal resolution are the detector and the tracer. The timing resolution (full-width at half-maximum of the instrument response function) of our detector (H7422P-40, Hamamatsu) is about 230 ps (Supplementary Fig. 12). Whereas this response time is typical in photon-counting detectors, it is still orders of magnitude faster than charge-coupled devices. It is well known that localization accuracy of a fluorescent particle relies heavily on the brightness and photostability of this fluorescent molecule^43^. Assuming that a bright fluorophore has an emission rate of 10^8^ s^−1^ and 10% of emitted photons are collected, the detected photons could exceed 10^7^ per second. As ∼100 photons are needed for particle position determination with moderate precision, this could mean that the ultimate temporal resolution for SPT is ∼10 μs. Recently, another group reported achieving 10 nm 3D localization precision and 10-μs temporal resolution in confocal 3D tracking of a giant quantum dot (QD) cluster (∼40 QDs)^18^. As our 3D tracking approach has better collection efficiency (non-descanned detection and the emission light is not split among multiple detectors), we see no potential problem for our system to reach similar temporal resolution and localization precision using a tracer system with ultrahigh emission rate but with a short lifetime.

Despite a shorter time gate of 3.3 ns, demultiplexing of longer lifetime emitters can be performed by applying a fluorescence correction factor in our tracking algorithm (further discussed in Supplementary Fig. 12). The correction factor allows tracking of lifetimes up to 4.5 ns by subtracting the estimated crosstalk from the previous time gate. The correction factor must be adjusted for fluorophores of different lifetimes. With a correspondingly smaller correction factor, we demonstrate tracking a short lifetime emitter, Cy5-tagged beads (1.18 ns), and show that lifetime can be accurately measured (±0.08 ns) during tracking (Supplementary Fig. 12). However, for extremely long lifetime emitters, such as quantum dots, or emitters whose lifetime changes dynamically, a lower repetition rate source or a pulse picker would be needed, in conjunction with longer physical delay lines.

Moving the beam through the sample instead of moving the sample itself^18^,^19^,^26^ has the additional advantage that beam steering is generally faster than the movement of a potentially heavy stage. Also, steering the beam is particularly suitable for applications in neuroscience, such as patching clamping, which requires stationary samples. While the stage response frequencies (10 kHz for the galvo mirrors and 300 Hz for the objective z-piezo stage) do not determine the temporal resolution of our tracking system, they impose an upper limit on the observable particle speeds^35^. The way to bypass this limitation in mechanical scanning and completely rule out the possibility that the observed dynamics are influenced by the objective motion (via mechanical coupling through the immersion medium) is to use fast adaptive optical elements in both lateral scan^26^,^44^ and axial focusing^45^.

An important characteristic of our tracking microscope lies in the fact that it is readily compatible with a number of fluorescence spectroscopy or microscopy techniques for probing molecular interactions, potentially at the true single-molecule level^46^, including fluorescence resonance energy transfer^47^ (via lifetime measurements), multicolour single-molecule imaging/spatiotemporal colocalization analysis^48^, step counting^49^,^50^, and mean-square displacement analysis^44^. Since only one detector is used for 3D tracking, additional detectors can be easily added and used for simultaneous, multicolour detection, which can provide a structural overview of the particle's surrounding environment or indicate molecular interactions. On the other hand, it is not straightforward to detect the second colour simultaneously on the traditional confocal tracking set-ups that employ 3–5 detectors for spatial filtering^18^,^19^,^20^, as twice as many detectors and serious alignment effort may be required for multicolour detection. The unique nature of the tetrahedral point spread function allows easily configurable beam spacing which may be optimized for measurements such as 3D pair correlation analysis^51^.

Here we demonstrate a new 2P 3D SPT microscope (TSUNAMI) that addresses fundamental limitation of deep and high-resolution SPT in the 3D space. Extended from our current tracking results in multicellular models, we are working towards direct *in vivo* 3D SPT at high spatiotemporal resolution. Our system will allow researchers to explore new questions in receptor transport and dynamic processes directly in 3D tissues.

## Methods

### 2P-3D-SPT instrumentation

The spatiotemporal beam multiplexer (Fig. 1) is created entirely from passive optics comprises beam splitters, mirrors and waveplates (Supplementary Fig. 1). A single-pulse train from a modelocked Ti:Al_2_O_3_ laser (Mira 900, Coherent) tuned to 835 nm is used as the primary beam, which then gets multiplexed into four beams offset in time and space. For temporal offsets, it was determined that to equally space four beams with an original 76 MHz repetition rate the delay time must be 3.3 ns, which corresponds to ∼1 m physical path length. For spatial offsets, each beam is first coaligned onto the primary optical axis. Adjusters that control *x* and *y* offsets are moved until a lateral spacing of ∼500 nm is achieved in the image plane. Alignment repeatability is verified by projecting fiduciary marks onto the laser scanning microscopy image during alignment such that each beam's centre is aligned to the same point in space to sub-100 nm repeatability. Axial spacing is controlled using a telescope assembly placed in the optical path of one beam pair to adjust their collimation. The alignment of axial spacing is verified using molecular detection function 3D scans (Supplementary Fig. 4) until a spacing of 1 μm is achieved (Supplementary Fig. 3). Following the optical multiplexer the beams pass through a galvo scanning system (6125H, Cambridge Technology), before being focused through a 60 × 1.3 numerical aperture silicone oil objective (UPLSAPO60X, Olympus).

For a typical experiment, we use an average laser power of ∼2 mW per beam (8 mW) total at the objective back aperture. For a typical φ40 nm fluorescent bead (F8770, Life Technologies.) photon count rates are in the range of 500–800 kHz (Supplementary Fig. 17) and targets can be tracked for durations up to 10 min (Supplementary Fig. 16). Background fluorescence signal is on the order of 3 kHz that includes a 150 Hz background signal from the detector. Signal-to-noise ratios are typically above 20.

### Demultiplexing emission signal

Demultiplexing of fluorescence emission is performed by time resolving the excitation contributions of each of the four multiplexed beams with fast photon-counting electronics. The fluorescence signal detected is an interwoven stream of photons excited by all four excitation beams. That signal can be time gated with 3.3 ns increments to effectively isolate the signal contributions from each beam and consequently isolate the signal contribution in space as well. Emission demultiplexing is performed electronically via TCSPC analysis. Fluorescence signals are detected by a cooled GaAsP photomultiplier tube with 5-mm square active area (H7422PA-40, Hamamatsu) in non-descanned configuration. The current output from the PMT is amplified through a 2 GHz cutoff bandwidth preamplifier (HFAC-26, Becker and Hickl GmbH) and sent into a photon-counting board (SPC-150, Becker and Hickl GmbH) to be counted and correlated to the 76-MHz reference clock of the laser oscillator. Given the electronics setup our fundamental timing resolution is on the order of the instrument response frequency, which was measured to be 230 ps full-width at half-maximum (Supplementary Fig. 12).

### Tracking control software

The tracking acquisition control loop is run entirely in LabVIEW (National Instruments) on the Windows 7 operating system. Time-resolved signals from the TCSPC board can be read into LabVIEW by two methods: (1) histogram mode and (2) First In, First out (FIFO) mode (also known as Time Tag). In histogram mode, the TCSPC board performs on board histogramming of the photons detected during a single-control loop period (5 ms) and sends the data to LabVIEW for processing into new control signals (Supplementary Fig. 2). In FIFO mode, each photon event is recorded by the board and processed on the fly in the LabVIEW control loop. For a loop rate of 5 ms and typical particle count rate of 500 kHz, this requires time binning 2,500 photons that are represented with 12-bit precision. This task is easily achievable with current computing hardware. The data on each photon event can be recorded for post processing and re-binning to achieve timing resolutions down to 50 μs with sufficiently bright particles (Supplementary Figs 11 and 18). Deterministic timing for the LabVIEW control loop is achieved by forcing the program to run on a hardware-timed clock from a peripheral component interface (PCI) data acquisition board (PCIe-6353, National Instruments). Using this hardware-timed loop method periods down to 1 ms can be requested with no missed cycles for up to 60 s. At 5 ms, the timed loop can run indefinitely with no missed cycles up to 20 min.

Control signals are generated by taking the ratio of the summed photon counts in each of the four time gates. Following the below formulas,


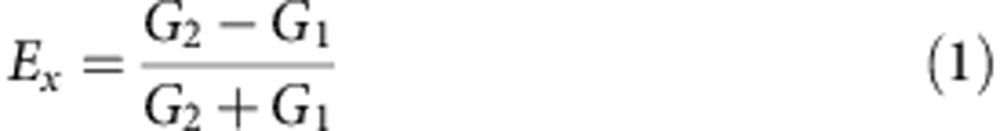



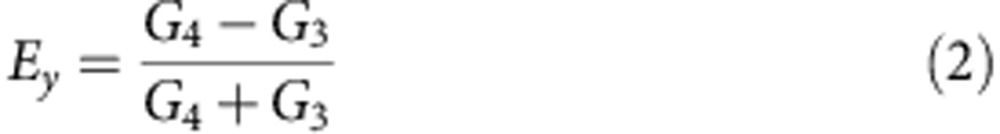



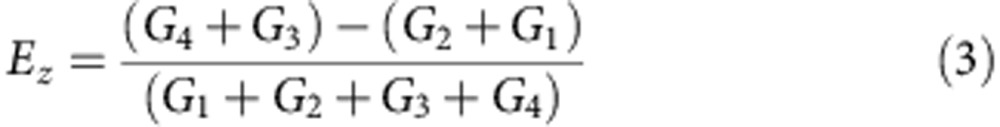


where G_1_, G_2_, G_3_ and G_4_ are the total photon counts in each time gate. Error signals *E*_*x*_, *E*_*y*_ and *E*_*z*_ are modified by a proportional controller before being sent out by the PCIe-6353 board as analogue signals to their respective actuators (galvos for *x* and *y*, and objective piezo stage (P-726 PIFOC, PI) for *z*).

### Sample preparation

EGFR-overexpressed A431 skin cancer cell was purchased from American Type Culture Collection and cultured in Dulbecco's Modified Eagle Medium (Cat. No. 11995-065, Life Technologies) supplemented with 5% fetal bovine serum (Cat. No. SH30071, Thermo Scientific). The cell cultures were kept in humidified atmosphere with 5% CO_2_ in air at 37 °C. Agarose-coated 96-well plates were used to cultivate A431 spheroids. The spheroids were prepared as previously described^52^ and the plates were incubated for 96 h in a humidified atmosphere with 5% CO_2_ at 37 °C. Cell-seeding density was 125 cells per well (Supplementary Fig. 10). Both monolayer cells and spheroids were kept for additional 24 h under serum-starvation condition before EGFR tracking. Plasma membrane was stained with CellMask and surface EGFRs were labelled with fluorescent nanoparticles (Supplementary Fig. 8). To label EGFRs, the cells were incubated with 1.5% bovine serum albumin solution (Cat. No. S7806) for 15 min at 37 °C, and then EGFRs were labelled with biotinylated anti-EGFR antibodies (200 ng ml^−1^ in 1.5% bovine serum albumin solution; EGFR Ab-3, Cat. No. MS-311-B, Thermo Scientific) for 15 min at 37 °C (Supplementary Fig. 9). The antibody solution was removed and cells were washed twice using PBS. The stock solution of φ40-nm fluorescent nanoparticles (FluoSpheres NeutrAvidin-Labelled Microspheres No. F8770, Life Technologies) was sonicated for 10 min and then diluted to 100 pM in DMEM. This solution was added into samples for 5 min at 37 °C.

## Additional information

**How to cite this article:** Perillo, E. P. *et al*. Deep and high-resolution three-dimensional tracking of single particles using nonlinear and multiplexed illumination. *Nat. Commun.* 6:7874 doi: 10.1038/ncomms8874 (2015).

## Supplementary Material

Supplementary Figures, Supplementary Methods and Supplementary References Supplementary Figures 1-18, Supplementary Methods and Supplementary References

Supplementary Movie 1 A high resolution 3D Trajectory of a single ø100 nm green fluorescent bead freely diffusing in an 80% glycerol mixture. The center of the particle is locked into focus using optical feedback from the four overlapping excitation beams. The box is 6×8×4 μm3 and the duration of the trajectory is 43 seconds.

Supplementary Movie 2 Directed motion trajectory, an independently controlled 3D stage to moved a single fixed particle through a known trajectory while the tracking microscope kept the particle locked into the focus. The spiral is 800 nm in diameter and 6 μm long with a duration of 5 seconds and 2 μm s^-1^ mean velocity. Localization root-mean-square error for x y and z is plotted in the graph to the right.

Supplementary Movie 3 Demonstration of 50 μs time resolution by resampling trajectories with photon event data (Supplementary Fig. 11). A ø40 nm red fluorescence microsphere was tracked in a solution of 50% wt. glycerol for 5 seconds (diffusion coefficient ~1.8 μm^2^ s^-1^). This movie shows a 0.5 second portion of that trajectory with both the original loop sampling time, 5ms, and the super-sampled reconstructed trajectory with 50 μs sampling. The bounding box dimensions are 1×1×2 μm^3^.

Supplementary Movie 4 Movie of main text Fig. 3. An example EGFR entry trajectory measured in a monolayer A431 cell culture. A single labeled EGF molecule (See fluorescence bead and EGFR description) was tracked for a duration of 460 seconds. The movie playback rate is 20 times. The cell size is approximately 30 μm × 10 μm. The cell membrane is plotted as a red iso-surface while the nucleus is a blue iso-surface. In the beginning of the trajectory the molecule is membrane bound and slowly moving ~0.4 μm s^-1^, at approximately 150 seconds the molecule is internalized as seen by the high speed (2 μm s^-1^) directed motion with a total displacement of 1 μm. Trajectory is rainbow colored with blue denoting the beginning.

Supplementary Movie 5 Movie of main text Fig 4. An EGFR trajectory measured in an A431 Spheroid model at a depth of 50 μm within the spheroid. A ±5 μm slice at depth 50 μm was taken with LSM, the spheroid outer plasma membrane is displayed in red iso-surface form while the nuclei are displayed as blue iso-surfaces. The total image size is 51×51 μm^2^. A single EGF molecule is tracked for 350 seconds, movie playback rate is 15 times. The trajectory starts on the plasma membrane and a high speed (~2 μm s^-1^) directed motion is observed ~250 seconds into the trajectory. The total displacement during internalization is 1 μm. Trajectory is rainbow colored with blue denoting the beginning.

Supplementary Movie 6 Movie of Supplementary Fig. 14. An EGFR trajectory measured in an A431 Spheroid model at a depth of 90 μm within the spheroid. This trajectory demonstrates slow transport modes and interaction with the nucleus. A ±5 μm slice taken with LSM, the spheroid outer plasma membrane is displayed in red iso-surface form while the nuclei are displayed as blue iso-surfaces. The total image size is 51×51 μm^2^. A single EGF molecule is tracked for 600 seconds, movie playback rate is 25 times. The trajectory starts on or near the plasma membrane and has most likely already been internalized. Over the course of 10 minutes the molecule moves slowly (~0.17 μm s^-1^) towards a nucleus with total displacement of 3.5 μm. The trajectory appears to follow the contour of the nuclear iso-surface image.
