# Supplementary material for: Deep and high-resolution three-dimensional tracking of single particles using nonlinear and multiplexed illumination
Source: Nat Commun. 2015 Jul 29;6:7874. doi: 10.1038/ncomms8874 (PMC4532916; doi:10.1038/ncomms8874)
Supplement: Supplementary Figures, Supplementary Methods and Supplementary References — Supplementary Figures 1-18, Supplementary Methods and Supplementary References [file ncomms8874-s1.pdf]

Supplementary Figures

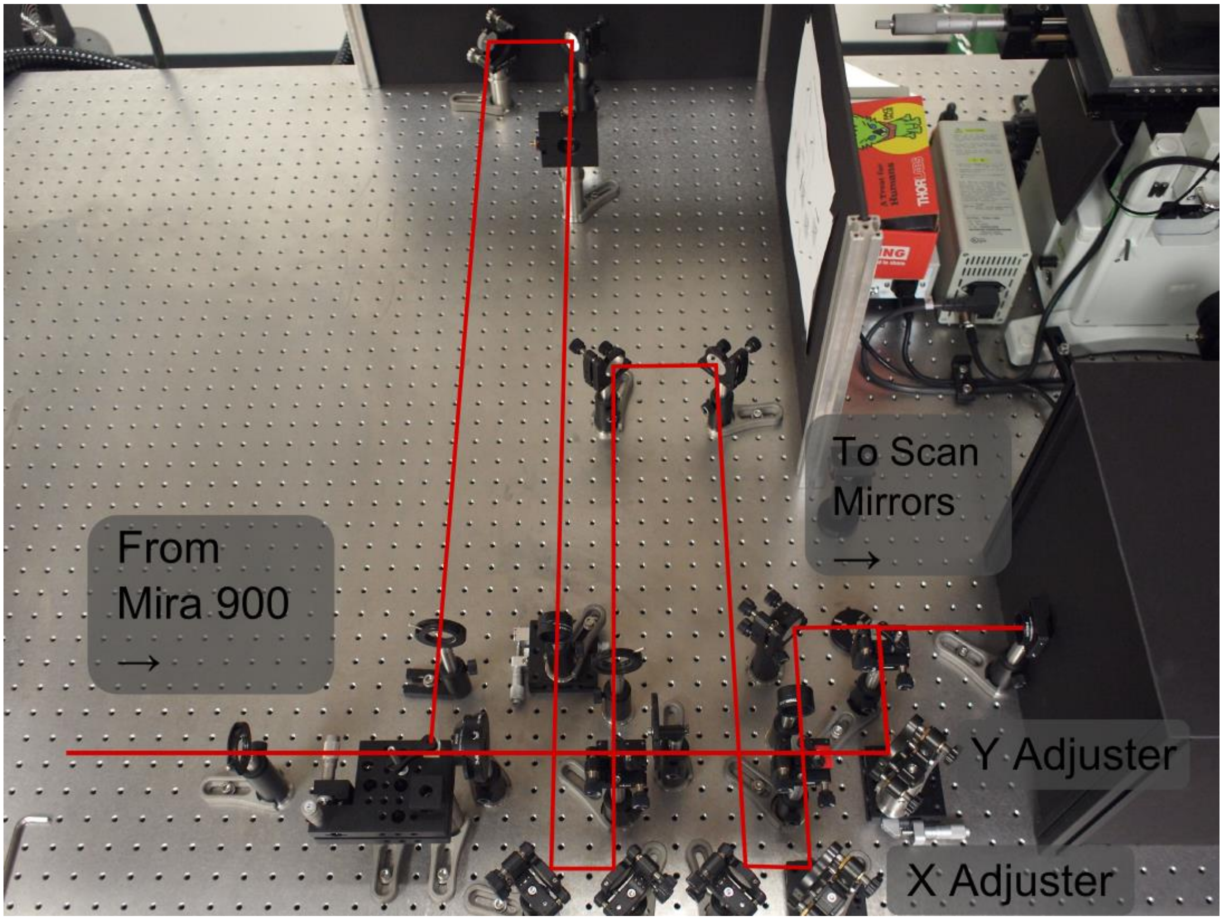

Supplementary Figure 1. TSUNAMI setup photo.
A photograph of the beam multiplexer optical system. A 76 MHz pulse train from a mode-locked Ti:Al_20_3 (Mira 900, Coherent) is split into 4 beams and recombined onto a single optical axis. Beam spacing is tuned by mirrors marked as x and y adjusters. The design is such that the offset adjustments are made as close as possible to the scanning mirror module to reduce lateral offsets in the beams at the microscope back aperture. Although the angular deviation between beams is small (100-200 arcsec) over a great distance the offsets lead to significant power imbalance from aperture clipping. Another design consideration was to minimize beam divergence over such large delay distances (up to 3 m for the fourth excitation beam). Divergence was mitigated by way of a 4× beam expansion using a telescope assembly prior to entering the beam multiplexer system. The beam diameter was expanded from 2 mm to 8 mm to reduce divergence proportionally. Following the multiplexer the beams are reduced back to 2 mm width with a telescope and relayed through a galvo scanning mirror system (6125H, Cambridge Technology). The relay design is such that the aperture of the beam multiplexer is imaged onto the galvo scan mirrors while the image formed by the galvo scan mirrors is relayed onto the back aperture of the microscope objective. If this condition is satisfied there will be minimal lateral shift on back aperture from both the image of the galvo scan and the image of the multiplexer.

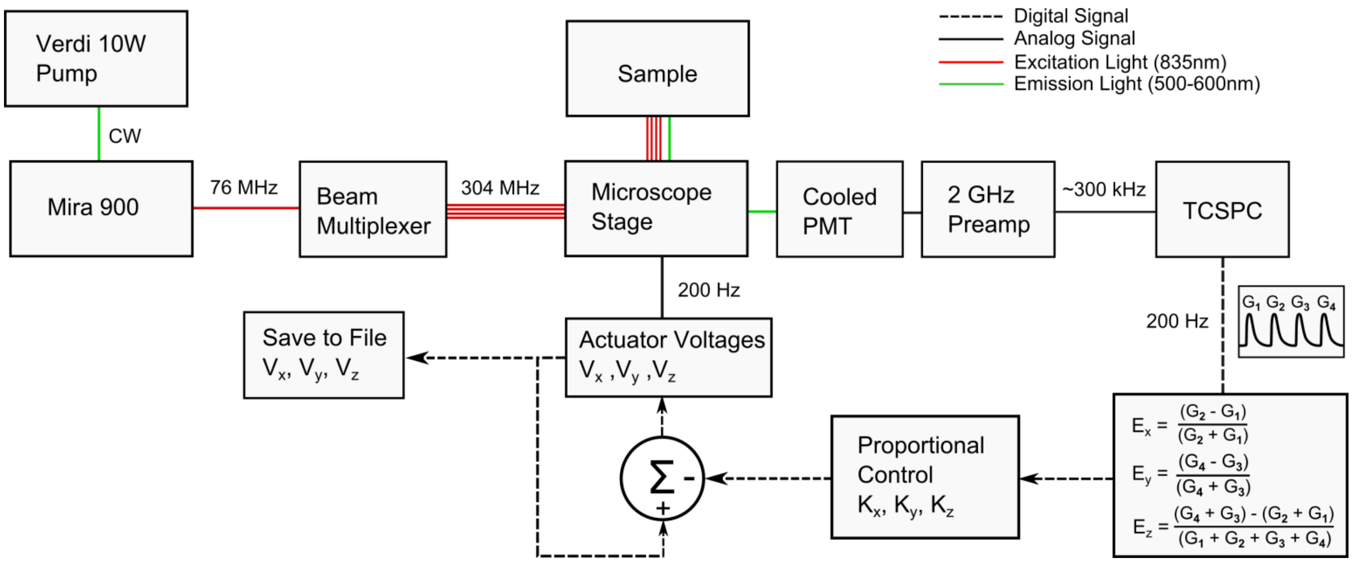

Supplementary Figure 2. TSUNAMI control schematic.

A Control Schematic showing system level interaction and feedback control loop. A free running 76 MHz pulse train generated by a Ti:Al_2O_3 oscillator (Mira 900, Coherent) passes through the beam multiplexer (**See Supplementary Methods**) and onto the sample. Fluorescence is detected by a cooled low dark count PMT (H7422P-40, Hamamatsu Corp.) and amplified with a 2 GHz cutoff bandwidth preamplifier (HFAC-26, Becker and Hickl GmbH). The amplified analog signal is then counted and correlated to the reference clock of the Ti:Al_2O_3 oscillator with a PCI-based photon counting board (SPC-150, Becker and Hickl GmbH). Every 1-5 ms a photon histogram is polled from the TSCPC module and processed in the software loop run entirely in LabVIEW (National Instruments). The tracking algorithm employs the simplest type of controller, a proportional control to convert the error signals to new stage positions. New voltages are sent out through a DAQ (PCIe-6353, National Instruments) to their respective actuators, scan mirrors for X and Y, piezo objective stage for Z.

Due to the time critical nature of this measurement technique it is imperative that the control loop operates at the desired frequency, typically 5 ms. To achieve this deterministic requirement in windows operating system the LabVIEW control loop was driven by an external hardware timed clock (counter from PCIe-6353 board) using this method deterministic loops can be achieve with rates up to 2 kHz without missing a hardware clock tick. For a typical 8 minute trajectory there are no missed loops out of 96,000 requested with a 5 ms (200 Hz) control loop period.

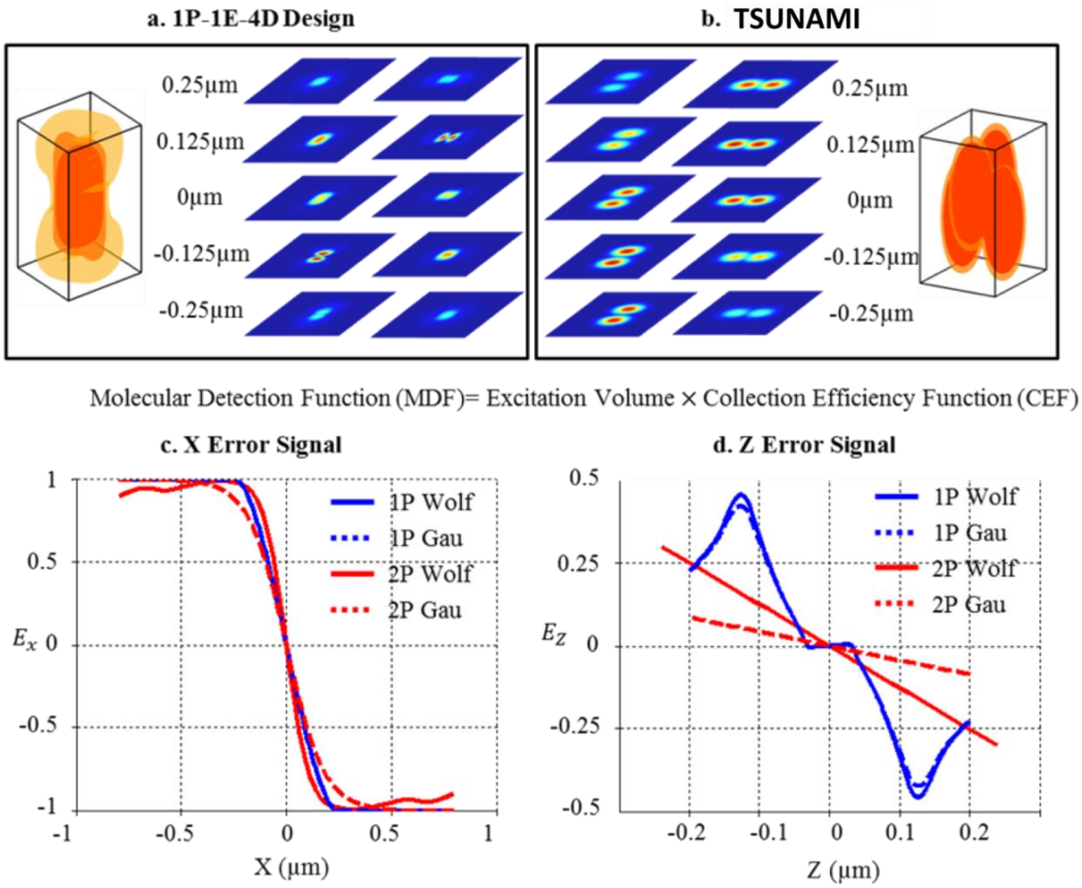

Supplementary Figure 3. 1P-1E-4D and TSUNAMI comparison.

a and **b,** Comparison of molecular detection functions (MDFs). **a.** In a specific embodiment of a 1Photon-1Excitation-4Detector design (a confocal 3D tracking microscope system invented by J. Werner and coworkers),[2] the calculation indicated that the spatial resolving capability of MDFs fades away at a distance of 0.25 μm from the center of the MDFs (located at ±0.125 μm), mainly due to the flattening of collection efficiency function (CEF) at this Z depth and a single excitation volume. As the spatial resolving capability disappears, target molecule will be lost. **b.** In the TSUNAMI design, the MDFs are dominated by the spatiotemporally multiplexed two-photon excitation volumes, as the PMT has a large active area (*i.e.* CEF~1). **c** and **d:** Error signals comparison of 1P-1E-4D (blue curve) and TSUNAMI (red curve). Solid lines: Error signals derived from the Wolf excitation field expressions. Dashed lines: Error signals derived from the Gaussian-Lorentzian excitation expression. E_y has the same dependence on y as E_x on x. The z-plane separation of both systems are set to be at 0.25 μm for comparison. The linear region of error signal is where the target molecule can be effectively tracked. E_x is steeper than E_z, meaning our tracking systems are more sensitive to XY displacement than Z displacement. As a result, the XY tracking accuracy is always better than the tracking accuracy in Z. In the specific 1P-1E-4D confocal tracking microscope design modeled the system has higher tracking sensitivity (specifically in Z) compared to TSUNAMI. However the increased sensitivity comes at the price of smaller lock-in range. Design factors such as fiber spacing can be optimized for specific performance metrics, although practically this becomes costly as new custom fiber bundles must be installed to achieve a different fiber spacing in X and Y. By contrast, the TSUNAMI system allows flexible control of the PSF because the spacing is performed through mirror actuators and not fixed fiber offsets.

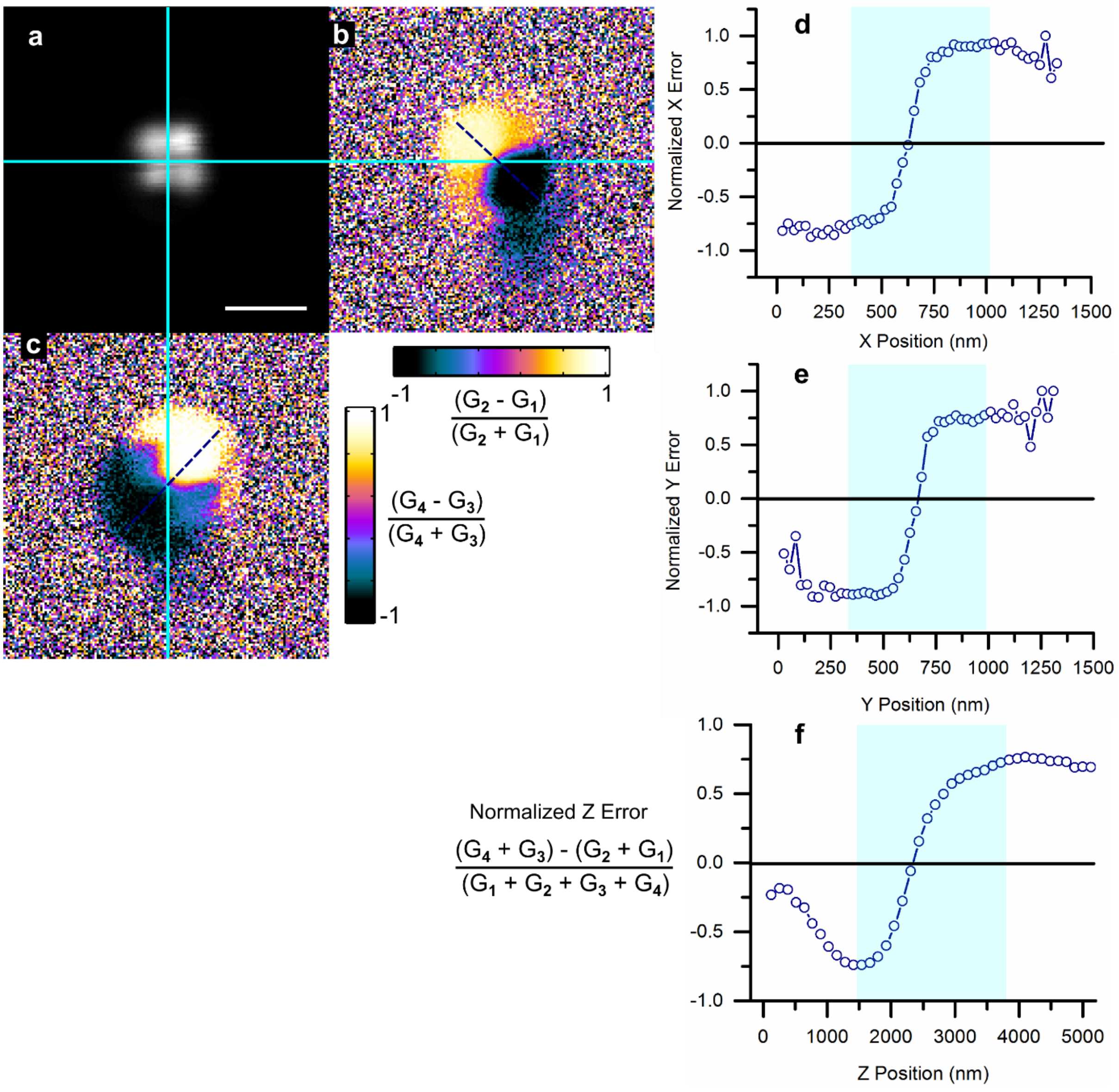

Supplementary Figure 4. Molecular detection function.

To calibrate our 3D localization technique ⌀100 nm fluorescent beads were immobilized in an agarose gel and imaged ~20 μm past the coverslip. 3D images were taken in 3 by 3 by 5 μm^3 stacks. At each voxel of the stack a photon counting histogram was recorded using 5 ms integration time. The raw waveform was demultiplexed (explained in **Supplementary Fig. 2**) and processed to yield error values along all three dimensions. **a.** The composite image of all 4 beams at the mean z focus. Blue lines denote the center x and y axes. **b.** The X error map which is a calculation of x error weight E_x at every pixel in the image. **c.** Y error map which is a calculation of the y error weight, E_y, at every pixel in the image. **d.** A line profile through the x error map at the location drawn in Fig. **b.** Precision target tracking is feasible over a 500 nm range. **e.** Normalized error line profile through y error map (Fig. **c**) showing near exact positional dependence on error as the x profile, as expected. **f.** Normalized error through all images in the stack located at the mean intensity position denoted by the intersection of the blue lines in Fig. **a**. To smooth the curve the error value was calculated with 2 adjacent pixels in each image, the slope remains the same. Scale bar is 1 μm.

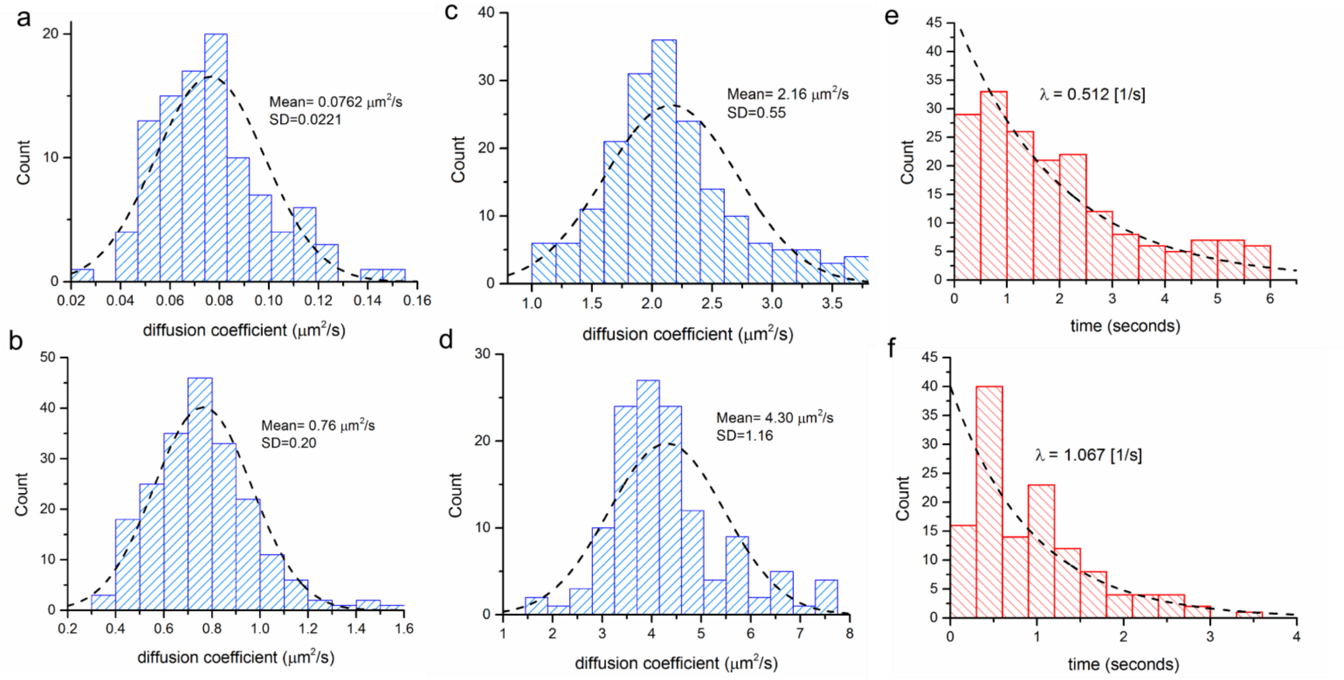

Supplementary Figure 5. Free diffusion validation with glycerol titration.

The system's capability to track particles under free diffusion was tested by observing mobile ⌀100 nm fluorescent beads in a range of different viscosities. The fluorescent beads were mixed with glycerol and water to achieve concentrations of **a.** 80% **b.** 50% **c.** 25% and **d.** 0% glycerol by weight. Particles were tracked for a duration of 10 seconds or until they were lost. Between 100 and 150 trajectories were taken at each data point. The diffusion coefficients are calculated by finding the mean-squared displacement for all time delays, $x(\tau)$, from the raw trajectory data,

$$6Dt = \langle (x(\tau) - x_0)^2 \rangle \ ,$$

The diffusion coefficient parameter, D, was extracted from a linear regression of the MSD vs. τ curve. A linear regression model is used because the particles are undergoing free diffusion.[6] Histograms of the measured diffusion coefficient recorded from different viscosities **(a-d)** all represented a normal distribution with central tendency that agreed well (within 1% at 21°C) with the theoretical Stokes-Einstein relation. **e.** Histogram of trajectory durations, with 25% glycerol mixture, which is found to follow an exponential distribution with a mean tracking duration of ~2 seconds. **f.** Histogram of trajectory durations with 0% glycerol and 100% water mixture. With glycerol mixtures above 25% wt. the target locking was stable and more than 99% of the trajectories were recorded up until the software imposed time limit of 10 seconds.

To avoid large standard deviation in the diffusion coefficient calculation, data from trajectories of duration less than 0.2 seconds were not recorded. This rejection criterion can be seen by the dip at zero value bin in the histogram in **Fig. f**.

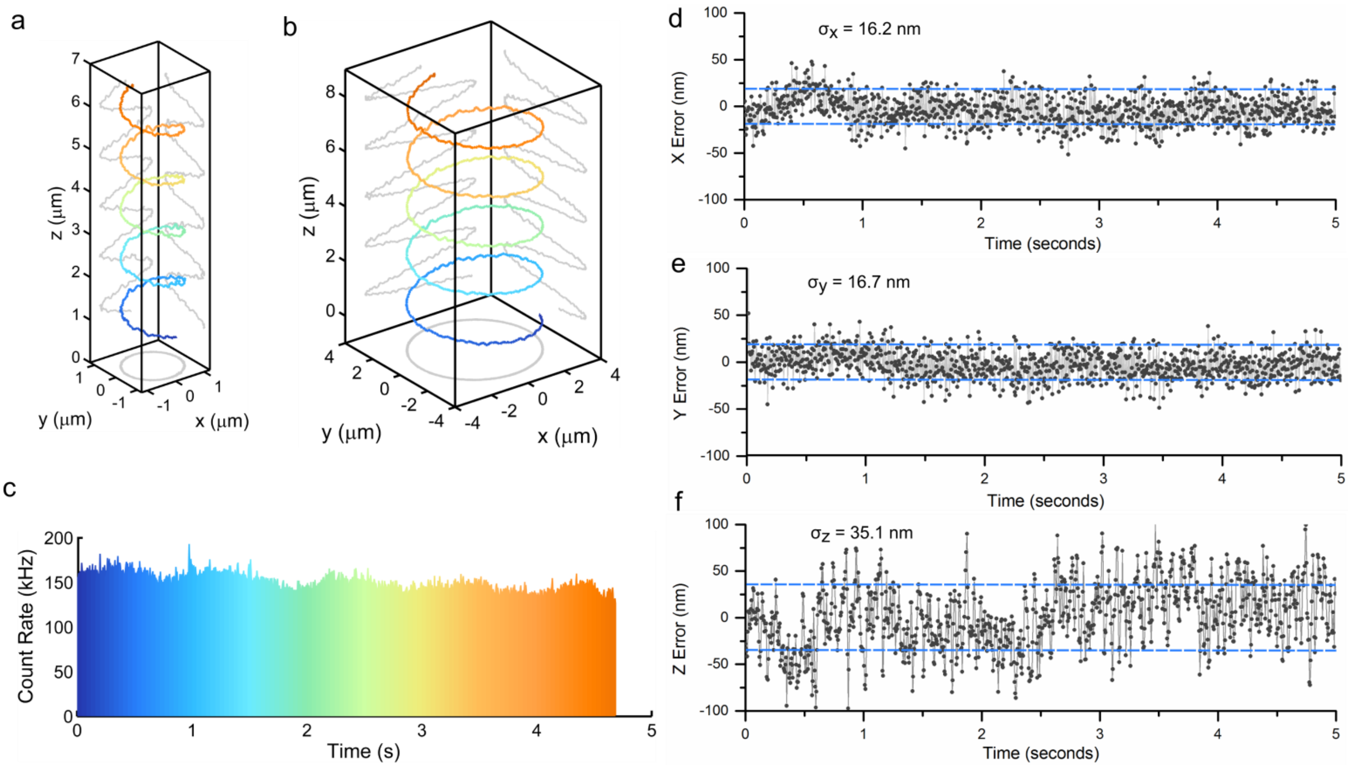

Supplementary Figure 6. Localization uncertainty measurements.

a. Additional helical trajectory of ⌀100 nm fluorescent bead (F-8803, Life Technologies) fixed to coverslip and scanned using 3D piezo stage. The scan was programmed for ±800 nm xy range and 6 µm s^{-1} mean velocity. **b.** Additional helical trajectory of ±3 µm xy range and 9.6 µm s^{-1} mean velocity. **c.** Particle brightness over time for trajectory in Fig. **a**. measured in photon counts per second. At high velocities the count rate oscillates due to a slight misbalance in the excitation efficiencies of each beam, this effect was only noticed for helical trajectories with mean velocities higher than 6 µm s^{-1}. Localization accuracy remains acceptable even for very high mean velocities up to approximately 10 µm s^{-1} where the particle displacement within a 5 ms time-step is too large for the proportional controller to remain locked into the target. **d,e,f.** Localization error graphs for trajectory in Fig. **a** along x, y, and z dimensions respectively. The error value at each time-point was calculated by the difference between the tracking actuator outputs (scan mirrors for xy, objective piezo for z) and the feedback sensor measurements in the 3d path scanning stage. With a sub-nanometer resolution it is assumed that the 3d stage feedback sensors represent the actual position of the stage in a given time point. The localization uncertainty was calculated from the raw error values by taking the root mean squared deviations (rms)[7,8]. The rms deviations of the error signal were 16 nm for x y and 35 nm for z even for high velocities up to 6 µm s^{-1}.

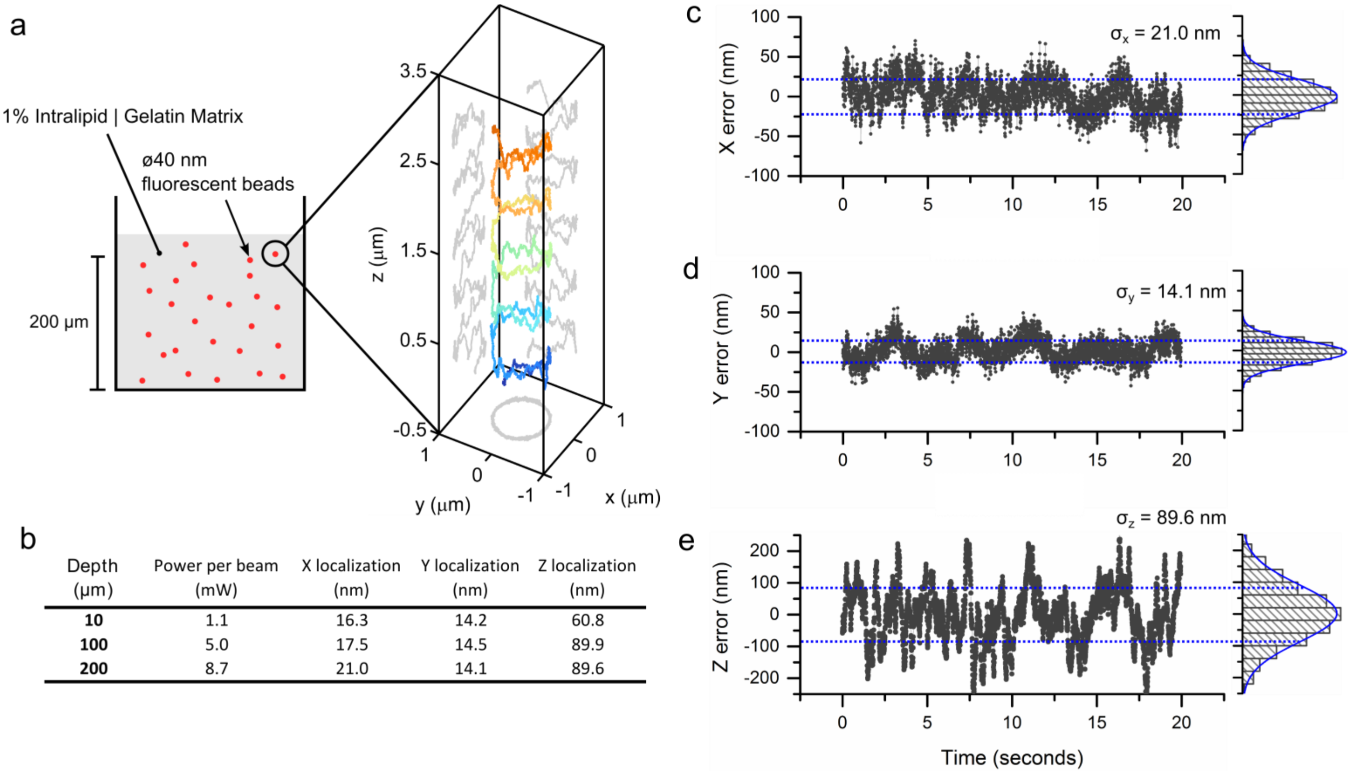

Depth (µm)	Power per beam (mW)	X localization (nm)	Y localization (nm)	Z localization (nm)
**10**	1.1	16.3	14.2	60.8
**100**	5.0	17.5	14.5	89.9
**200**	8.7	21.0	14.1	89.6

Supplementary Figure 7. Localization uncertainty through turbid media.

a. ⌀40 nm fluorescent beads (F-8770, Life Technologies) were prepared in a matrix of 1% Intralipid and 9% gelatin to mimic a turbid tissue sample. Directed motion was performed as described previously (**Supplementary Fig. 6)** with an independently controlled 3D piezo stage **b.** Helical directed trajectories were measured at three different depths all with a mean velocity of 1.0 µm s^{-1}. Trajectories were recorded at 10 µm, 100 µm, and 200 µm past the coverslip. As expected required excitation power increases with increasing depth, while localization accuracy decreases. X and Y localization suffer only a minor decrease, from ~16 nm to ~21 nm rms whereas the Z localization experiences a slightly increased reduction in precision from ~60 nm to ~89 nm rms. This reduction in Z localization can be attributed to an elongated PSF that occurs when light is focused through scattering samples that effectively blurs the beams along z and lowers the optical contrast signal required to lock onto the target. **c,d,e.** Localization error values recorded at 200 µm depth for x,y, and z dimensions respectively. Histograms are displayed to the right for each curve. It can be seen that these distributions can be fit very accurately to a normal distribution indicating that there are no systematic errors or instrument artifacts corrupting the localization even at a depth of 200 µm into the scattering sample.

I. Supporting Data

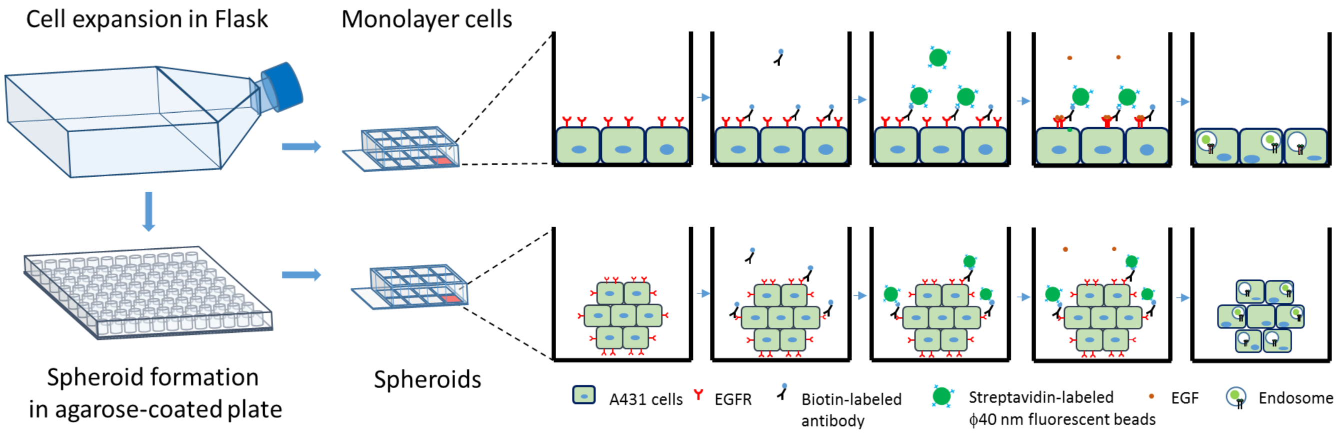

Supplementary Figure 8. Preparation of samples for EGFR tracking.
A431 cells were expanded in flasks and the dissociated into single-cell suspension with trypsin treatment. For EGFR tracking in monolayer cells, the cells from suspension were directly seeded into chamber slides. The monolayer cells were stained with CellMask™ Deep Red and their EGFRs were recognized by antibodies and labeled with fluorescent nanoparticles. For spheroids, the suspended single cells were seeded into agarose-coated and incubated for 96 hours to form spheroids. After serum starvation, the spheroids were transferred to chamber slides for membrane staining and EGFR labeling.

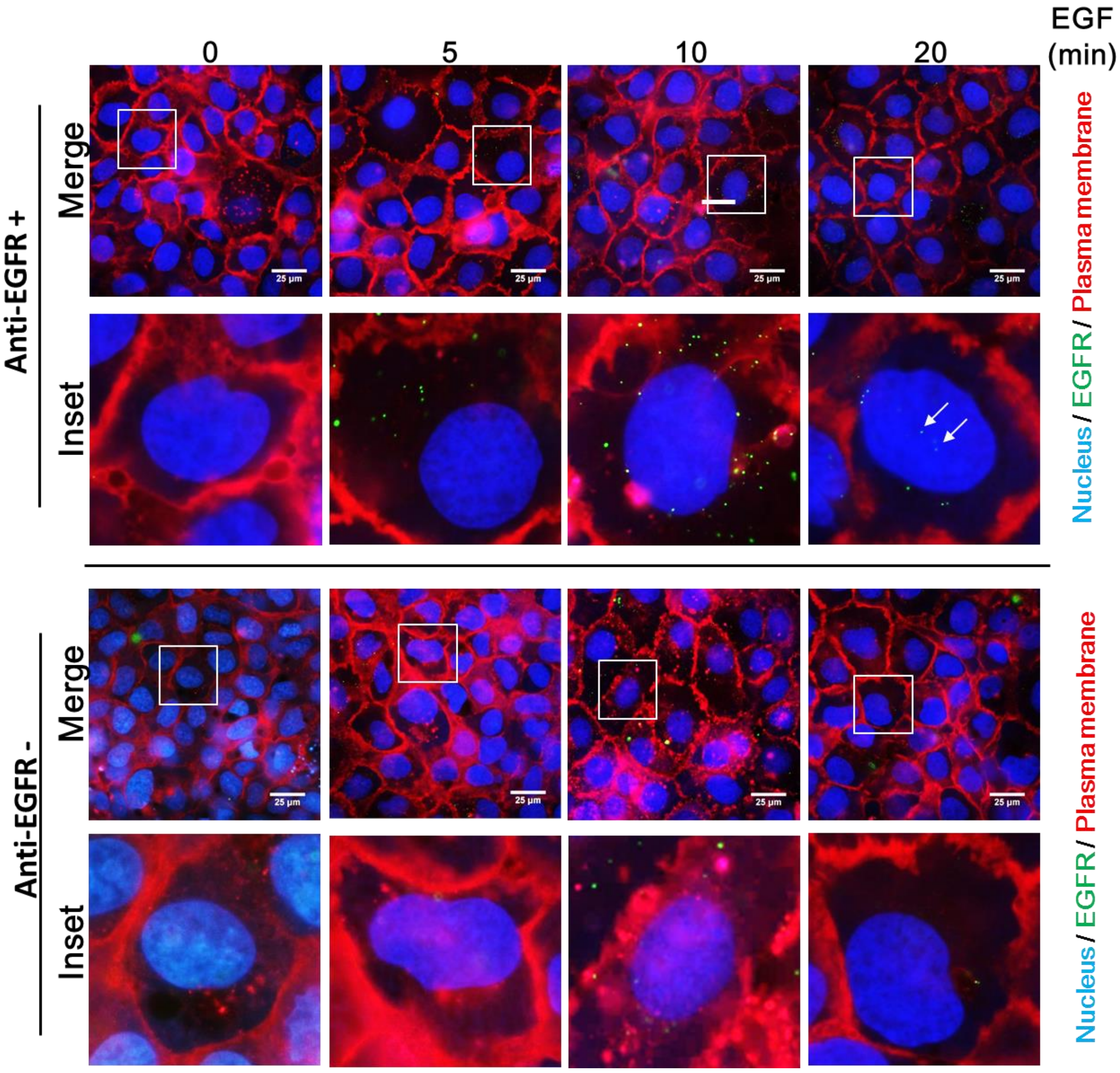

Supplementary Figure 9. EGF induces internalization of EGFR.

Monolayer A431 cells were exposed to serum-free media overnight, and then their EGFRs were recognized with biotin-conjugated anti-EGFR antibodies, and NeutrAvidin® conjugated red fluospheres (F8770, Life Technologies) bound to biotins to label EGFRs. In control group, we didn't label EGFR with anti-EGFR antibodies. After labeling, cells were treated with EGF (20 ng ml^{-1}) for indicated time. The white arrows indicate nuclear translocation of EGFR. The boxed areas are shown in detail in the insets. Scale bar is 25 μm.

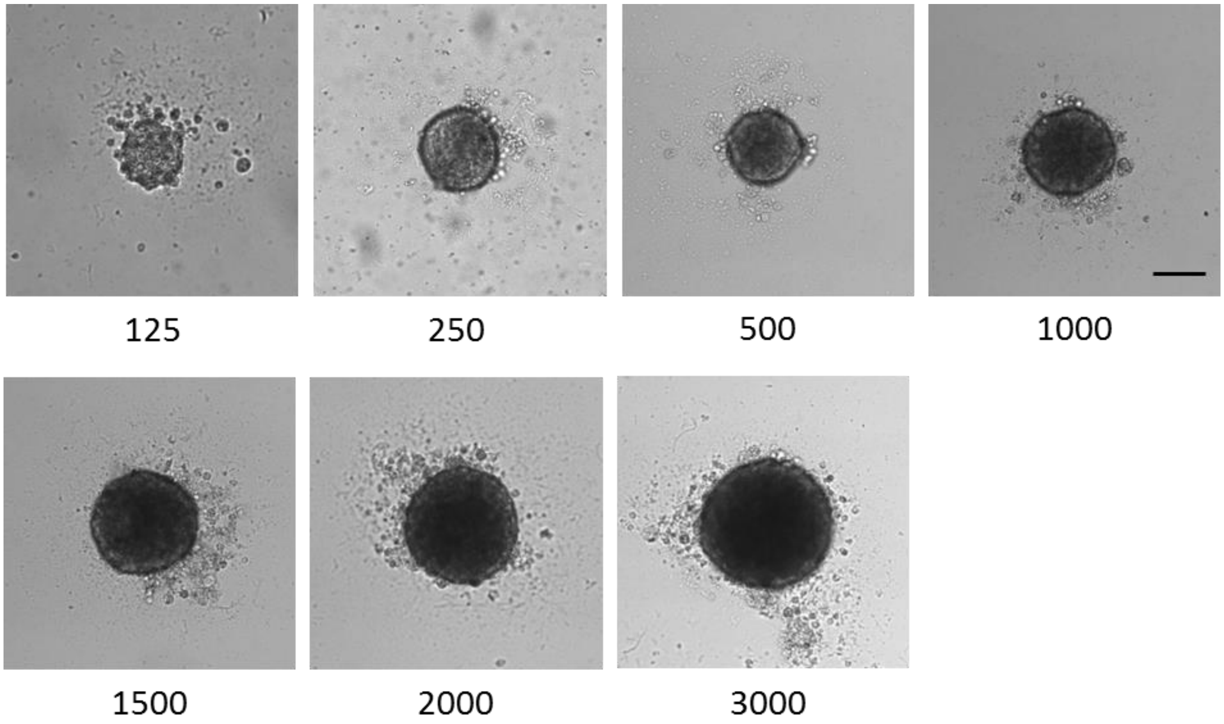

Supplementary Figure 10. Spheroid formation and initiation protocol for A431 cells.

Bright field imaging of MCTSs formed in liquid overlay from dissociated, exponentially growing A431 skin epidermoid carcinoma cells after a 96-hr initiation interval in agarose-coated 96-well microliter plates. The seeding density was between 125 and 3000 per well in 200 µl of serum-conditioned high glucose standard medium. The concentration to routinely and reproducibly obtain spheroids with a diameter of 90-110 µm is 125 cells per well. Scale bar is 100 µm.

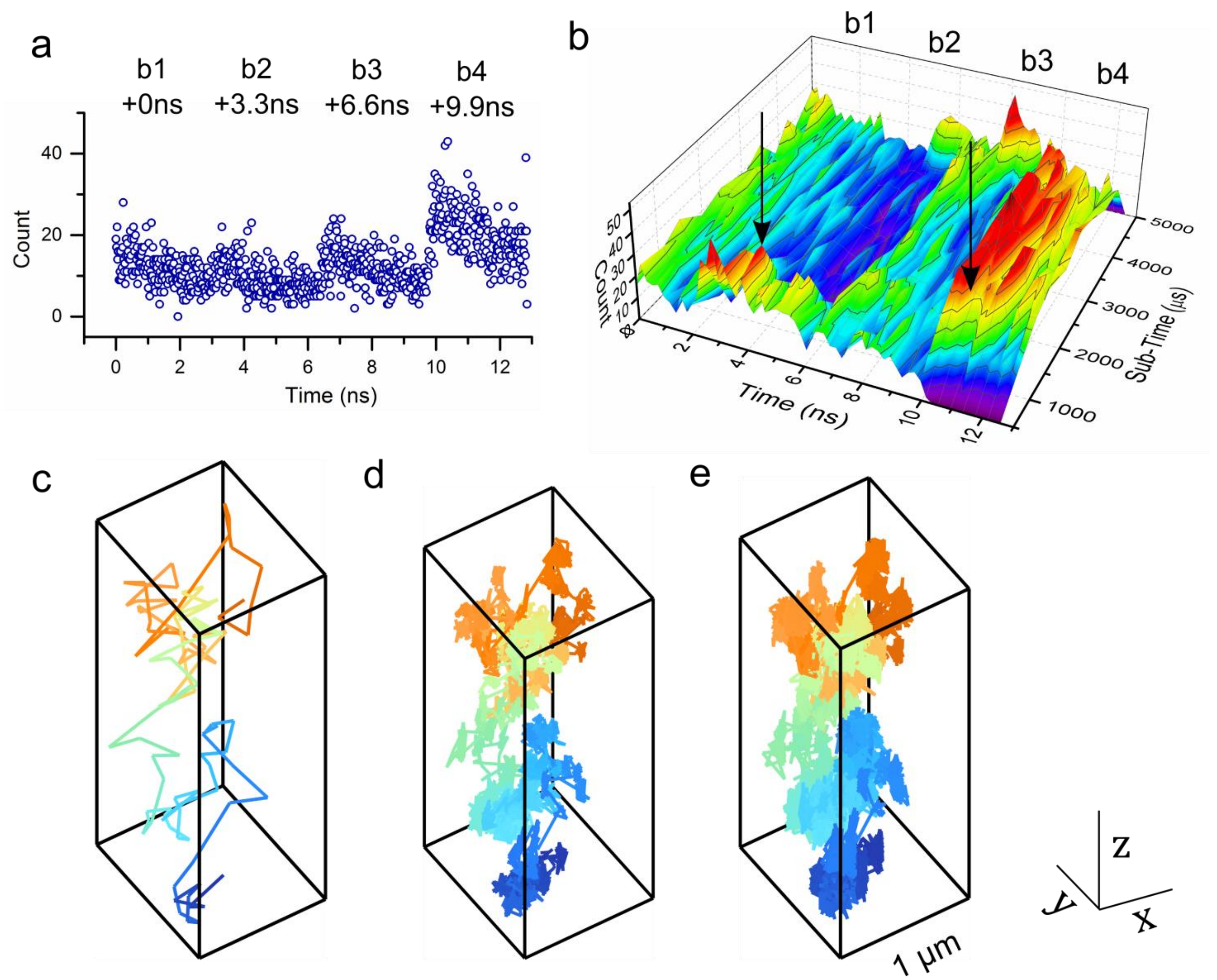

Supplementary Figure 11. 50 μs time resolution.

To demonstrate resolution down to 50 μs bright nanoparticle trajectories were measured and reconstructed using individual photon count data (**See Online Methods**). ⌀40 nm fluorescent microspheres (F8770, Life Technologies) were tracked in a 50% wt. glycerol solution under free diffusion. The individual photon count data stored in the FIFO was saved and analyzed in post-processing to reconstruct trajectories with higher temporal sampling. Using sufficient laser power (3 mW per beam) a photon count rate between 1-2 MHz was achieved, which corresponds to between 50-100 photons per localization at 50 μs time resolution. We have no problem to localize the particle with this number of photons as useful signal counts for each beam are 12-25 photons whereas background noise, even in the worst condition, is only 0.5 photons per beam (for 40 kHz background during live cell imaging). **a.** Example photon count histogram sampled from a single control loop period of 5 ms. **b.** The same photon count data resampled with 200 μs time bins and displayed as a 2D surface, x-axis is the micro-time in nanoseconds and y-axis is the resampled histogram time with 200 μs resolution. The black arrows denote a sudden shift in beam intensities resulting from the particle movement during the 5 ms integration period. A perfectly centered and stationary particle would appear as four ridges along the y axis of the 2D surface. **c,d,e.** A 0.5 second long trajectory sampled with 5 ms, 100 μs, and 50 μs time resolution, respectively.

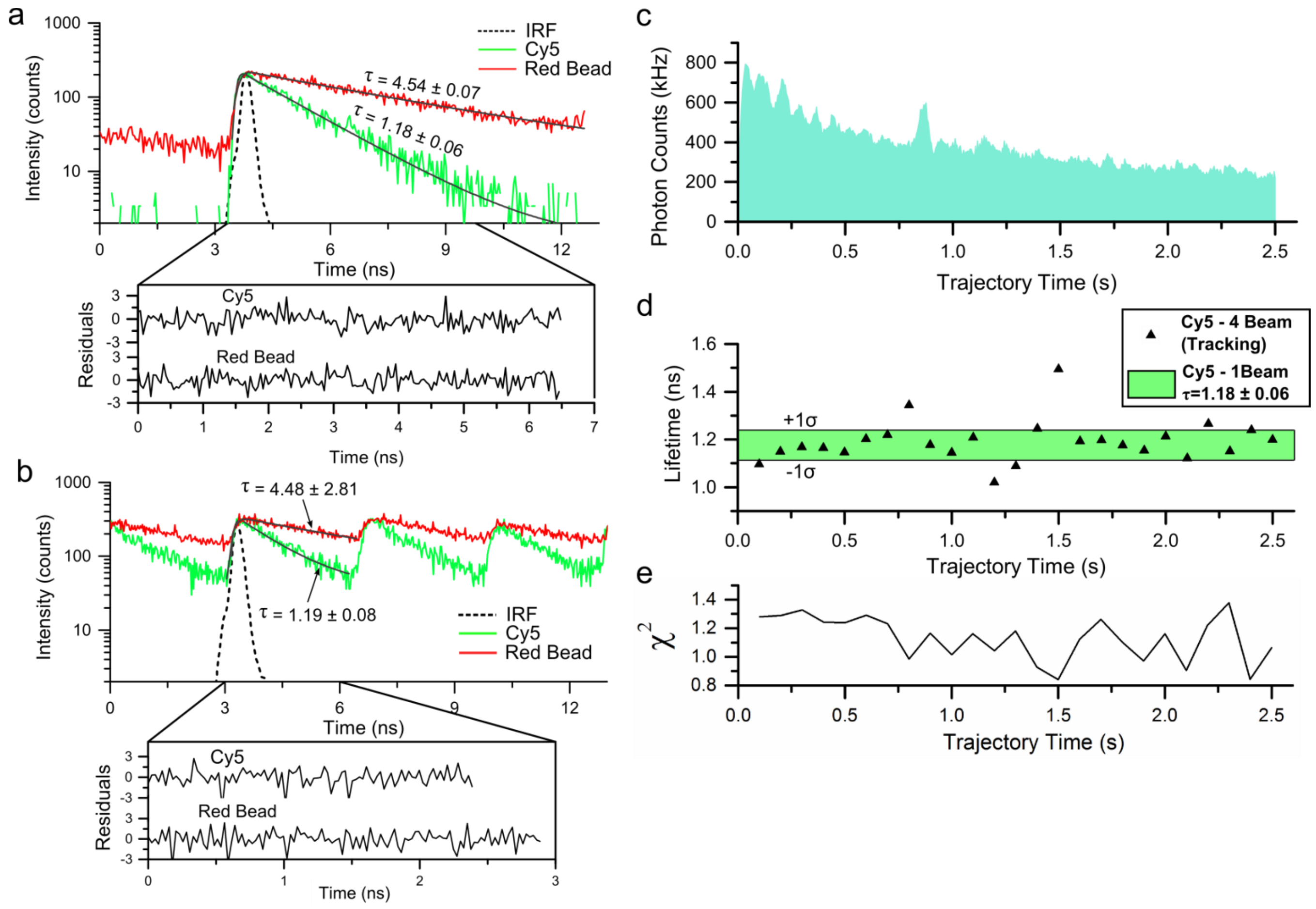

Supplementary Figure 12. Fluorescent lifetime measurement during tracking.
Fluorescence lifetime can be simultaneously recorded during tracking by using every photon arrival event as described in **Supplementary Fig. 11**. **a.** Single beam excitation and **b.** 4 beam excitation measurements using time-correlated single-photon counting (TCSPC) with two different fluorophores, Cy5 labelled microspheres (⌀500 nm, PS500-S5-1 NANOCS) and red fluorescent microspheres (⌀40 nm, F8770, Life Technologies). Both excitation regimes were performed using the same laser power, gain, and concentrations (40 pM) in 40% Glycerol solution. For single excitation measurements 5 decays were recorded and separately fitted. The average lifetime of the Cy5 microspheres under single beam excitation was 1.18 ns. This data was used as a reference from which to measure lifetime accuracy with four excitation beams. A commercial fluorometer (FluoTime 300, PicoQuant) was also used as a reference measurement. Four beam excitation measurements were performed while tracking, the reported lifetime is an average of 25 separately fitted data points from a 2.5 second trajectory (lifetime bin of 100 ms and tracking loop period of 5 ms). Fitting was performed using a least-squares minimization method with a Gaussian convolved exponential model,[9] the window size for fitting was the same for all data points in each trajectory. **a. and b. expansion graphs** show residuals for each fit within the windowed region.

c. Photon counts for the 2.5 second Cy5 bead trajectory. Even for low excitation power (<1 mW total) photo-bleaching is rapid due to surface conjugation of the dye onto the microsphere, an oxygen scavenger could be used to reduce bleaching an extend trajectory length. **d.** Resulting lifetime fits from a Cy5 bead trajectory (triangles) plotted against the lifetime measured using only 1 beam excitation showing that the results are statistically similar. 75% of the lifetimes calculated from tracking fall within 1 standard deviation of the Cy5 reference data.

Even with a 3 fold decrease in photon count signal, from 750 to 250 kHz, the Cy5 bead can be simultaneously tracked and report an accurate and consistent lifetime. The calculated lifetime was found to be 1.19±0.08 ns which agrees with both the reference Cy5 measurement (1.18±0.06 ns), and the fluorometer (1.11±0.01 ns). **e.** Chi squared values from the fit for each data point in the trajectory.

From comparing single beam and four beam excitation it is clear that tracking short lifetime fluorophores (< 2 ns) such as Cy5 is feasible, the lifetime extracted from tracking consistently matched reference measurements. However for fluorescence decays longer than the time gate (>3.3 ns) such as red fluorescence beads, the tracking data is inconsistent. The lifetime from tracking red beads was found to be 4.48±2.81 ns, compared with 4.54±0.07 ns for a single excitation reference. The mean is close to the reference; however the standard deviation is two orders of magnitude higher. The measurement inaccuracy for long lifetimes arises primarily from fluorescence crosstalk in each of the four time gates. It should be noted that, although crosstalk is an issue for lifetime measurements, it has only a minor effect on tracking stability and accuracy even for long fluorescence decays greater than the time gate. During tracking a fluorescence correction factor is applied to subtract the contribution from each of the previous time gates. Since the tracking localization algorithm is based on a simple intensity ratio and proportional control the effect crosstalk can be removed with almost no loss in localization or controller stability. By contrast, least-squares based lifetime fitting methods are highly sensitive to signal artifacts,[10] and so applying a similar correction factor to measure fluorescence lifetime does not yield an accurate result. Methods to overcome this limitation for lifetime measurements using this tracking system would be to extend the time gate using a lower repetition rate laser source in conjunction with longer physical delay lines, or to investigate computational methods for deconvolution of the TCSPC signal with four excitation beams.

The dead-time of the TCSPC board can result in pulse pileup artifacts. Typically this artifact is only a concern for high emission rates (enough to interfere with the dead-time). Our highest photon detection rate for 1 MHz count rate and 76 MHz laser repetition rate is 0.013, it has been shown that pulse pile-up effects will affect PMT measurements when the detection rate is above ~0.1.[11] Our highest detection rate is still 10 times less than the threshold for dead time and pulse pileup effects to become significant, so we believe our system has no artifacts introduced at our current range of emission rates with our TCSPC system.

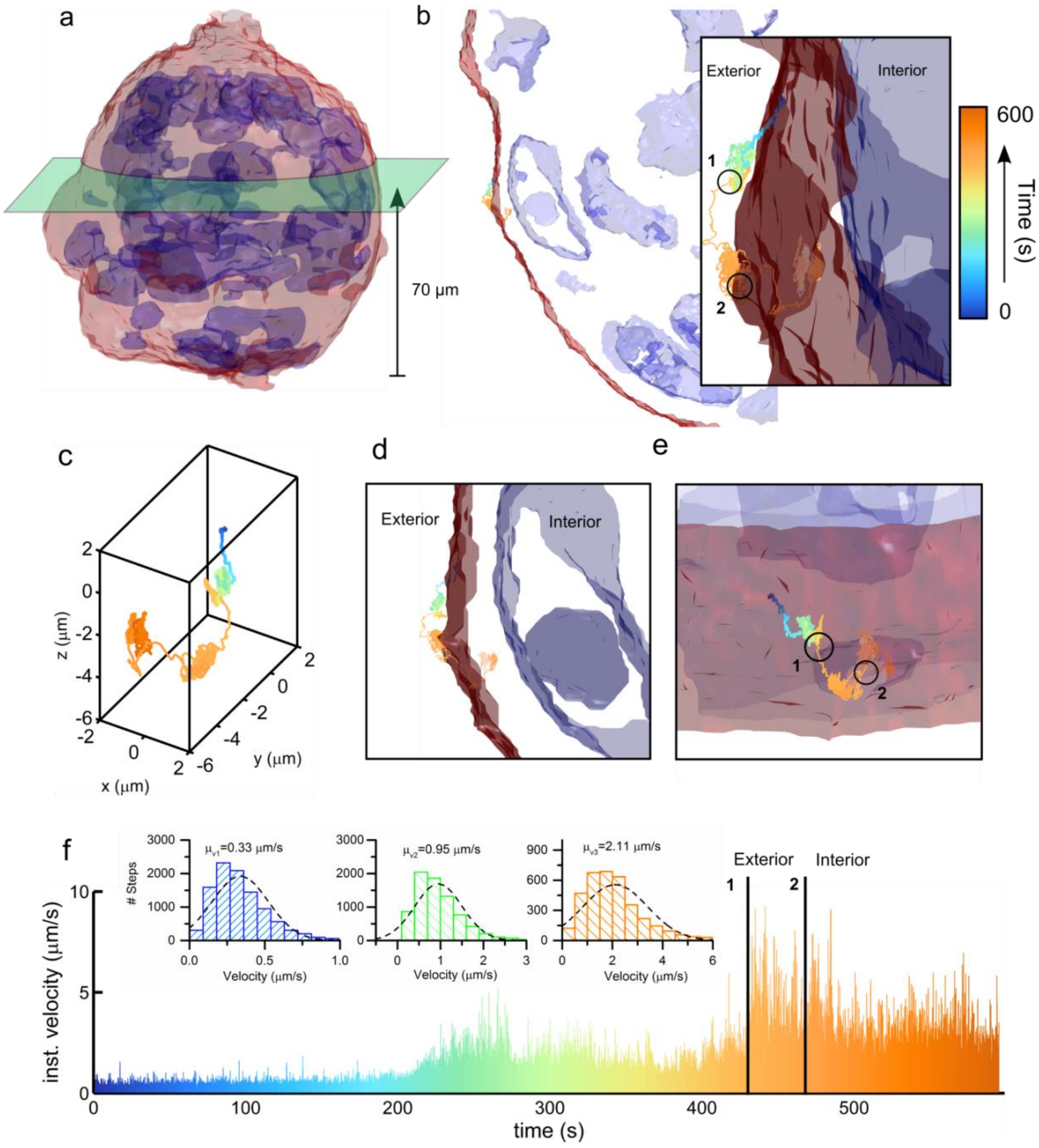

Supplementary Figure 13. Deep single particle tracking of EGFR in spheroid model.

An example EGFR entry pathway measured with 2P SPT 70μm deep in a spheroid model **a**. 3D Iso-contour of a ⌀100 μm spheroid taken with 2P LSM staining for plasma membrane (red) and nuceli (blue). The highlighted slice denotes the z-plane where the trajectory was measured. **b**. Close up 3D isocontour plot of a slice 70 μm deep into the spheroid taken with 2P LSM. (Inset: Close up view of the trajectory showing is location realtive to the plasma membrane). The trajectory is 10 minutes long, color coded by time, with blue denoting the beginning. Points marked 1 and 2 are regions with an onset of high velocity. Point 2 denotes the point where the particle was most likely internalized due to large displacement (~1μm) transverse to the membrane. **c.** 3D Trajectory plot with no cell overlay. **d.** Top view of the trajectory **e.** Side view of the trajectoy **f.** velocity plot color coded by time showing a stepwise increase in instantaneous velocity over the course of 10 minutes. (Insets: Histograms of regions of the instantaneous velocity plot. The particle starts at 0.33 μm s^{-1} mean velocity from time 0-100s, and accelerates to 2.11 μm s^{-1} from time 400s-500s.)

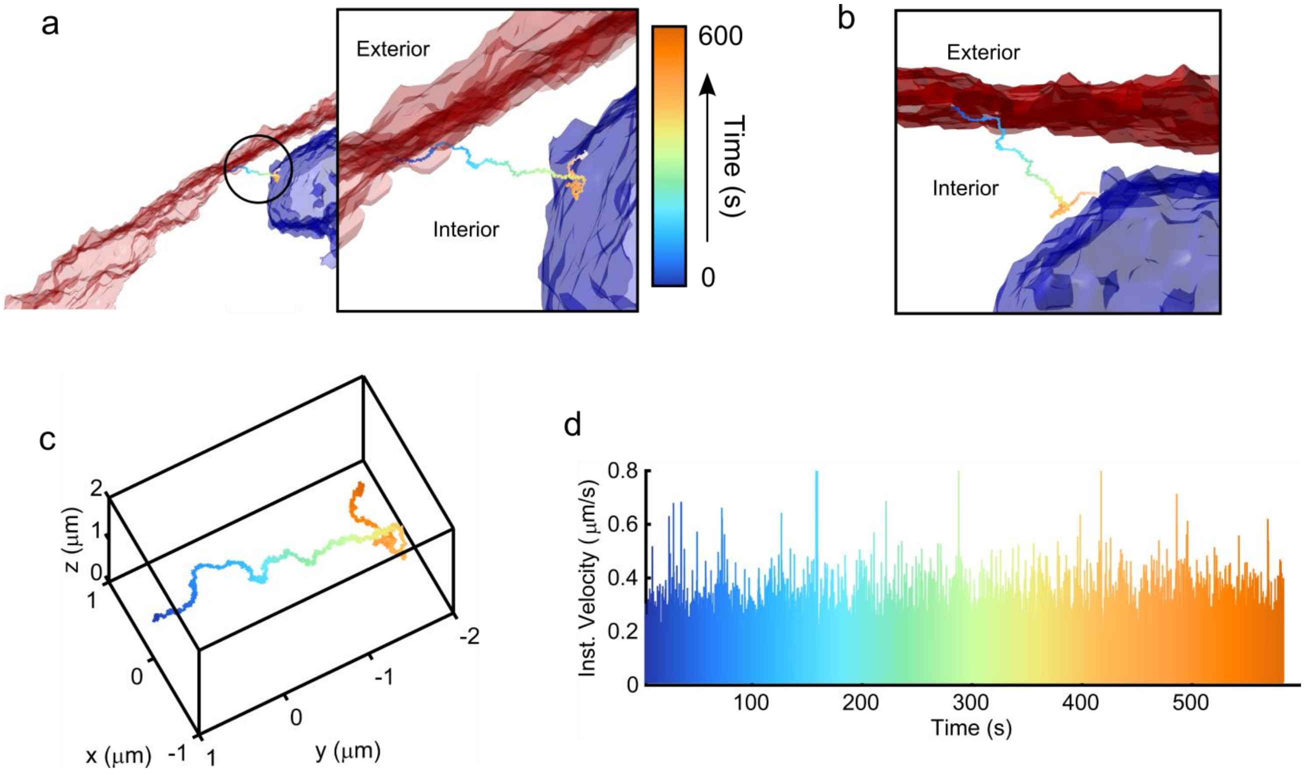

Supplementary Figure 14. Intra-cellular transport in spheroid model.

An additional EGF trajectory taken in a spheroid model. This example trajectory shows slow diffusive transport in cytosol and interaction with the nucleus. **a.** Isocontour of the zoomed in image stack taken 90 μm deep in a spheroid with cell membrane (red) and nucleus (blue) are overlaid with the trajectory (black circle), (inset: zoomed view of the trajectory). The trajectory begins inside the cell close to the plasma membrane and ends at the nuclear membrane. **b.** Additional close view of the trajectory from top down perspective. **c.** Plot of the trajectory. **d.** Instantaneous velocity plot over the 10 minute duration of the trajectory.

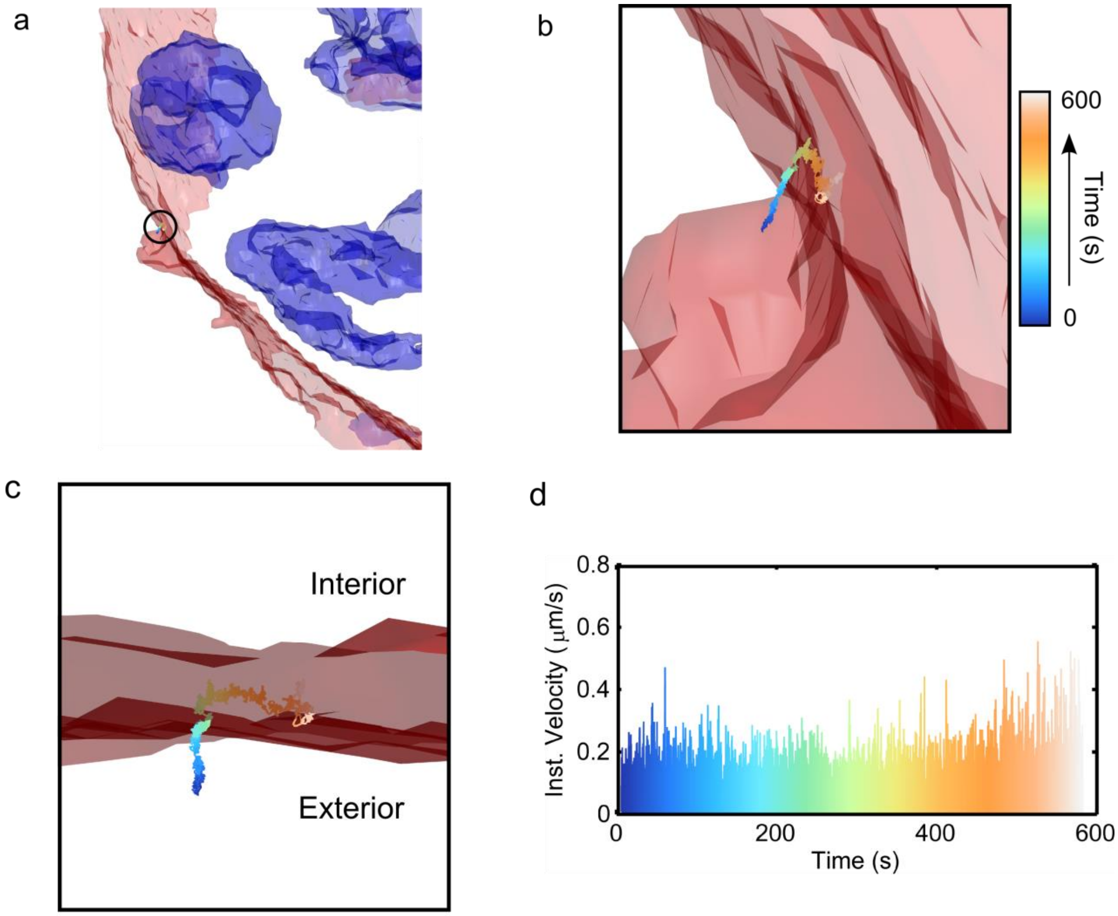

Supplementary Figure 15. Inhibited spheroid trajectory.

An example EGF trajectory with sodium azide inhibitor in a spheroid model. **a.** Isocontour image with cell membrane (red) and nucleus (blue) are overlaid with the trajectory (black circle) **b,c.** Zoomed images of the trajectory. Over the entire 600 second trajectory the displacement was ~600nm and no entry through the membrane occurred. **d.** instantaneous velocity plot with mean velocity 0.2 $\mu m\ s^{-1}$.

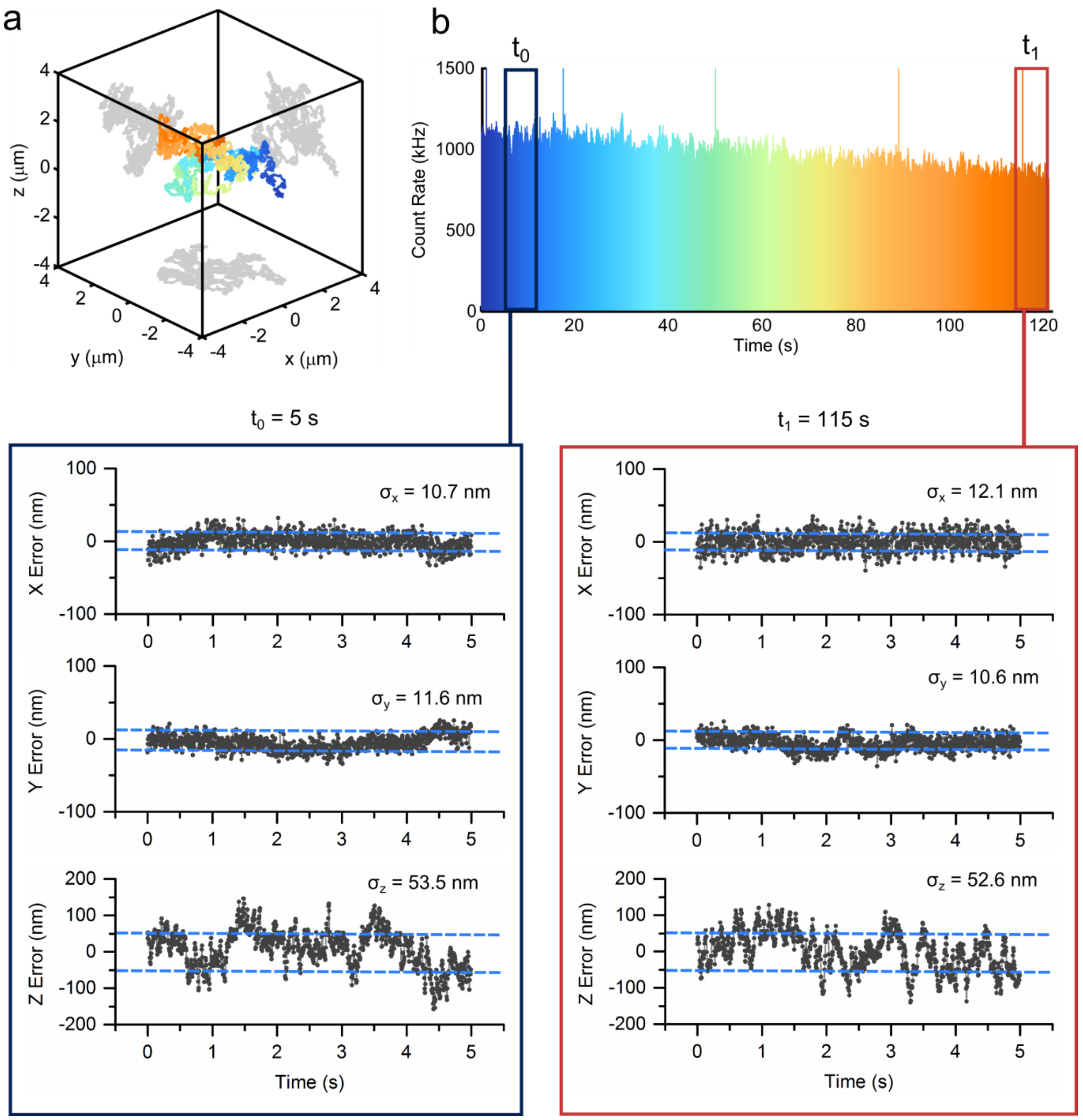

Supplementary Figure 16. Localization precision versus time.

This control experiment demonstrates a negligible decrease in localization accuracy over long time scales (>10 seconds) with decreasing number of signal photons detected. **a.** A ⌀40 nm fluorescent bead (F-8770, Life Technologies) was immobilized in gelatin and moved in a prescribed motion as described in **Supplementary Fig. 6.** The particle trajectory was simulated Brownian motion with diffusion coefficient of $0.05 \mu m^2 s^{-1}$ and duration of 120 seconds. **b.** Graph of photon count rate versus time. Over the 120 second trajectory the particle brightness decreased by ~20%. However, the localization accuracy was essentially unchanged with 10-12 nm in XY and 53 nm in Z. Typical 10 minute live cell trajectories have a brightness decrease of ~50%. Even with decreased brightness over a 10 minute trajectory the detected photon numbers are in the range of 1000 to 2000 for a 5 ms loop period, **Supplementary Fig. 18** demonstrates high resolution tracking (< 20 nm xy) with localizations using as low as 500 photons.

The initial count rate of the ⌀40 nm red fluorescent beads used for all live cell experiments is higher than that of the example count rate graph from **Fig. 2** primarily due to a further optimization of the microscope alignment and

excitation wavelength. The laser was tuned for optimal excitation of the red beads (830 nm based on internal calibration) which is not optimal for the ⌀100 nm green beads (a fluorescein analog)[12]. To achieve a similar SNR for the ⌀100 nm green beads requires higher excitation power and results in ~10 times faster photobleaching compared with ⌀40 nm red beads. However, this has minimal effect on the quantitative data used for the ⌀100 nm green bead trajectories, all of the experiments were shorter than 5 seconds, over which, there is negligible photobleaching (<20%) and minimal loss in localization accuracy (See **Supplementary Fig. 18**).

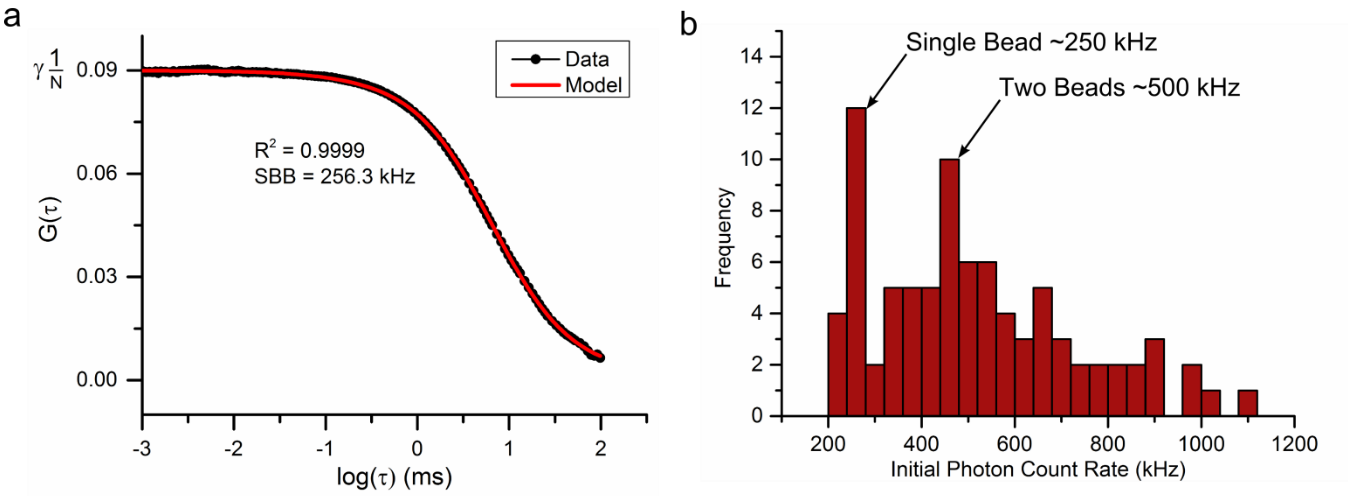

Supplementary Figure 17. Single bead brightness determined by FCS.

To estimate the number of beads attached to EGFR molecules during live cell experiments we characterize average single-bead brightness (SBB) using **a.** FCS Experiments and **b.** Trajectory analysis. FCS was performed with ⌀40 nm fluorescent microspheres (F8770, Life Technologies) in 16 nM concentration with a single excitation beam at 3 mW average power. Laser power and detector gain settings were matched to live cell experimental conditions. Raw photon counts were auto-correlated in real-time using a digital correlator (7002/USB, ALV). Autocorrelation curves (black dots) were averaged from 20 runs of 10 seconds each. To verify FCS quality, the autocorrelation data was fit to the following model (red line),[13] assuming a Gaussian-Lorentzian geometrical distribution ($\gamma = 3/16$).[14]

$$G(x) = \gamma \frac{1}{N}\left[\frac{1 - F + Fe^{-x/T}}{1 - F}\right]\left[1 + \frac{x}{\tau}\right]^{-1}\left[1 + \frac{x}{\alpha^2\tau}\right]^{-0.5} + DC$$

Where F (fraction of triplet state molecules) is 0.019, γ /N (average number molecules in detection volume) is 3, τ (dwell time in laser focus) is 6.519 ms, and T (triplet state relaxation time) is 100 µs. The model fit very well with an R^2 of 0.9999, the resulting single bead brightness was 256.3 kHz for a single excitation beam.

Further verification of SBB is done by analyzing 95 individual trajectories' count rates. **b.** Histogram of trajectory count rates at early time points in a fixed sample of ⌀40 nm fluorescent beads suspended in agarose. The initial count rate was taken to be the first 5 seconds of the trajectory after the controller had stably locked onto the particle (~100 ms). The histogram uncovers two peaks of brightness surrounded by a wide distribution ranging from 200 kHz to 1.1 MHz. The first peak is most likely the single bead brightness of 255 kHz whereas the second peak, at ~500 kHz, is likely an aggregate of two beads.

The SBB characterized by FCS, 256 kHz, is very similar to the direct measurement from 3D Tracking, 255 kHz. However FCS uses one excitation beam, whereas tracking is done with four, one would expect nearly a factor of four increase in signal relative to FCS if all excitation beams were collinear. During tracking the particle is not completely centered in the excitation volume of each of the four beams (xy offset ~500 nm, z ~ 1000 nm), this geometric offset is the primary source of discrepancy between FCS and Tracking SBB measurements. The trajectory SBB data, along with supporting FCS data, indicates that in the majority of live cell and spheroid experiments the system is tracking either single bead (~250 kHz) or two beads (~500 kHz).

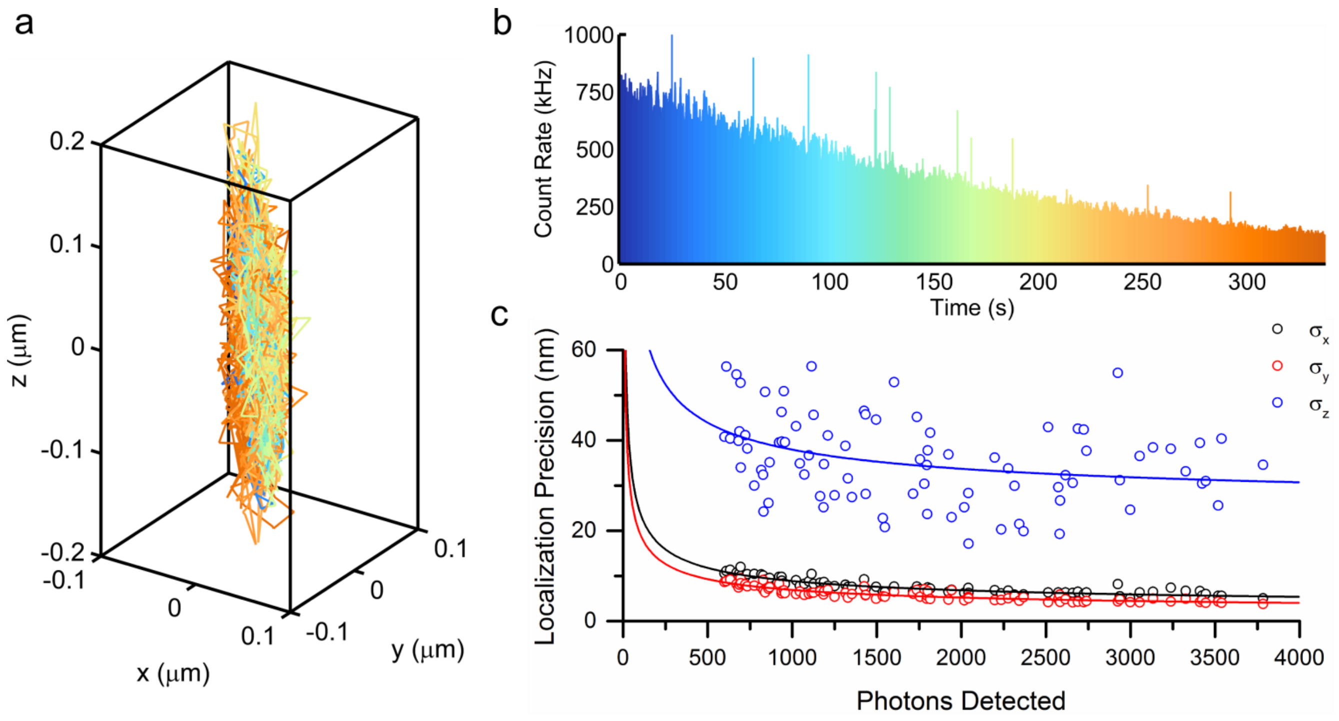

Supplementary Figure 18. Photon-limited localization precision.
A sample of ⌀40 nm fluorescent red beads fixed in agarose was used to evaluate localization precision as a function of number of detected photons. **a.** Trajectory of a fixed bead 20 μm past the coverslip tracked for 350 seconds. **b.** photon count graph. Laser power was increased (5 mW per beam) compared to live cell imaging (2 mW per beam, **Fig. 3**) to increase photobleaching rate and capture the entire range of detectable signals (750 kHz to 100 kHz). **c.** Scatter plot of localization accuracy in three dimensions for every time point in the trajectory, plotted as a function of detected photons (using a 4.5 ms collection time). A sliding window of 2 seconds width was used to generate the graph, dependent axis values are the mean photon count over the window. Fitting was done with a power function of the form, $\sigma \propto N^{-1/2}$.

Here a fixed bead sample was used as opposed to directed motion to eliminate any potential artifacts from an independent motion stage, and simplify the data analysis. The localization precision for a fixed bead is ~50% better than in the dynamic case (>2 μm s^{-1}) for a given photon signal (**Supplementary Fig. 5**) however the power law relationship still remains true in both cases demonstrating that this localization process is photon-limited with an approximate cutoff at 500 detected photons per loop period (i.e. 100 kHz). The z axis suffers from increased localization uncertainty, due to xy spatially variant error function. Ways to improve upon this would be to use MLE based estimators[15]. Future work will be to improve the z localization to reach a photon-limited precision.

It should be noted that for live cell experiments the photons used for localization starts in the range of 2000-4000 photons and over 10 minutes decays to ~1000-2000 photons. Therefore, it is expected that a loss of accuracy of approximately 30% occurs over a typical live cell trajectory period. Generally, the localization accuracy will remain better than 20 nm in xy and better than 50 nm in z over the entire trajectory.

Supplementary Methods

A setup photo and detailed description of the optical system for 3D particle localization can be found in **Supplementary Fig. 1**, the high level system block diagram **Supplementary Fig. 2** explains the control algorithm used to achieve high time resolution tracking capable of resolving dynamics down to 50 µsec. Discussion of particle localization characterization can be found in **Supplementary Fig. S4** and **Supplementary Fig. 6** for static and dynamic localization respectively. **Supplementary Fig. 11** explains the method used to achieve 50 µs temporal resolution.

Overview of 3D tracking technique. The demonstrated technique, coined as **TSUNAMI** (**T**racking **S**ingle-particles **U**sing **N**onlinear **A**nd **M**ultiplexed **I**llumination) achieves sub-diffraction limit localization with respect to a single emitter by evaluating an optical contrast function between 4 excitation beams positioned in a tetrahedral shape. Optical contrast is formed by slightly overlapping each excitation volume so that illumination from each beam on the target is spatially dependent, by taking the ratios of the quantified brightness (photon counts) due to each beam the emitter's location can be estimated using calibration curves (**See Supplementary Fig. 4**). This technique is similar to super-resolution localization techniques and prior 3D particle tracking work[1,2]. The unique multiplexed beam point spread function is formed through entirely passive optical elements placed before the scanning module of a standard two-photon microscope. To actively track a molecule the excitation tetrahedron is scanned across the sample using galvo scan mirrors, and the objective focal plane is scanned with a piezo stage. The optical outputs form a closed loop proportional control system run in LabVIEW on the windows 7 operating system.

Beam multiplexer optics for 3D tracking. Beam multiplexing is performed through an entirely passive optical system which splits a single ultrafast (~150 fs) pulse from a Ti:Al_2O_3 oscillator (Mira 900, Coherent) into 4 discrete beams that each have a unique spatial and temporal offset applied (**See Supplementary Fig. 1**). First, the original beam is split into two by a 50:50 non-polarizing beamsplitter (BS005, Thorlabs). The reflected beam is delayed by 6.6 ns over a 2 meter path length and passes through a telescope assembly using a singlet pair (Thorlabs) to adjust collimation. Meanwhile the transmitted beam passes through a half waveplate (AHWP05M-980, Thorlabs) to covert from p-polarization to s-polarization. Both the delayed p and non-delayed s polarized beams are split a second time into four beams by another 50:50 beam splitter, BS2. A second line delays the reflected beams by 3.3 ns and reflects off of an adjustment mirror, M1, before passing into a polarizing beam splitter, PBS (5812, Newport Corp.) The polarizing beamsplitter mixes the two beam pairs so that a second orthogonal offset adjustment can be made using mirror M2. All of the beams are recombined to a single axis using a third 50:50 beamsplitter, BS3. One arm of BS3 is not used so the light is blocked by a beam dump. After BS3 the beams are passed through standard two-photon laser scanning optics and into the microscope objective. Protected silver mirrors (PF10-03-P01, Thorlabs) are used throughout the system to fold beams onto the optical axis with minimal group velocity dispersion (GVD). Relative beam power is controlled with reflective neutral density (ND) filters (Thorlabs). ND Filters must be adjusted as wavelength is tuned but typically less than 10% difference in beam power is achieved for any wavelength and excitation differences are accounted for in the calibration procedure.

Feedback control. (Supplementary Fig. 2) shows the closed loop control schematic. The controller uses only a proportional gain to convert calculated error signals to galvo and piezo stage drive voltages. The optimal proportional gain was determined through the directed motion tracking experiments (**Supplementary Fig. 6**). The gain values were selected to minimize the rms positional deviations over the entire range of velocities. A single

set of gain values work well for the range of observable directed velocities, although it is possible to tune the controller to operate at higher average velocities, the tracking becomes unstable.

Beam multiplexer alignment procedure. Regular alignment maintenance can take only 10 minutes and is required once a day before an experiment. A typical daily alignment involves imaging of fixed nanoparticles using standard two-photon laser scanning imaging (LSM). When all 4 beams are illuminating the sample there will be 4 spatially offset images in the scan plane formed which can be used to align the beam spacing to sub-diffraction limited accuracy ~100 nm. Beam spacing is aligned using this laser scanning technique, to achieve repeatability we digitally display fiduciary marks on the image to guide the user in aligning each beam to its calibrated position on screen.

Two-photon laser scanning microscope. Excitation light is provided by a Ti:Al_2O_3 oscillator operating at 835 nm. (Mira 900, Coherent) The excitation light for laser scanning imaging shares the same path as the non-delayed beam in the multiplexer, the other 3 beams are blocked with shutters during laser scanning. After traversing through the multiplexer described in **Supplementary Fig. 1**, the light is sent through a galvo scan mirror pair (6215H, Cambridge Tech.) and expanded through a telescope assembly consisting of a 45 mm focal length achromatic doublet (AC254-045-B, Thorlabs) and a 200 mm focal length singlet (LA1979-B, Thorlabs). Following beam expansion the light enters the back port of an IX-71 Microscope stand (Olympus) and reflects off a dichroic mirror (750DCSPXR, Omega) before entering the back aperture of the objective (UPLSAPO60X, Olympus). The emission light is collected through the same objective and transmitted through the dichroic filter to be condensed onto three PMTs in a non-descanned configuration. The emission light is split into three color bands by two dichroic mirrors before being condensed onto each PMT (H7422PA-40, Hamamatsu). The first dichroic mirror picks of the blue-green spectrum with a reflection edge at 550 nm (FF552-Di02-25x36, Semrock) the second dichroic splits the remaining light into orange and red components with an edge at 640nm (FF640-FDi01-25x36, Semrock). Each PMT has a bandpass filter specific to the fluorescent targets used for the experiment, 460-500nm for nuclear stain (Hochest), 570-61 0nm for red fluorescent microsphere EGF-complexes (F8770, Life Technologies) and 680-720nm for membrane stain (Cellmask™ Deep Red, Life Technologies). The mirror scanning and data acquisition is handled by a single DAQ card (PCIe-6353, National Instruments) with a custom code interface in LabVIEW. Pixel dwell times are 2.6 microseconds which corresponds to a frame rate of approximately 1.4 Hz. Image sizes are typically $102 \times 102\ \mu m^2$ for whole spheroids, and $51 \times 51\ \mu m^2$ for monolayer cells and zoomed spheroids.

Cell culture. Used as the model system, EGFR-overexpressed A431 skin cancer cell was purchased from ATCC and cultured in DMEM (Dulbecco's Modified Eagle Medium, Cat. No. 11995-065, Life technologies) supplemented with 5% FBS (fetal bovine serum, Cat. No. SH30071, Thermo scientific). The cell cultures were kept in humidified atmosphere with 5% CO_2 in air at at 37ºC as previously described[3]. Single suspensions were prepared by mild enzymatic dissociation using a trypsin/EDTA solution. For EGFR tracking in monolayer cells, A431 cells were seeded onto optical imaging 8-well chamber slides (Nunc™ Lab-Tek™ II Chamber Slide™, Cat. No. 154534, Thermo Scientific) with the cell density of 1×10^5 cells per well and allowed to adhere overnight. In spheroid model, A431 cells were seeded into agarose-coated 96-well plate with the cell density of 125 cells per well for 96 hours incubation (See **Supplementary Fig. 10**). Both monolayer cells and spheroids were used for EGFR tracking in live cells.

Spheroid preparation. Agarose-coated 96-well plates were used to cultivate A431 spheroids. Each well of a 96-well plate (Cat. No. 130188, Thermo Scientific) was coated with 50 µL sterilized agarose solution (1.5% (wt. per

vol.) agarose (Cat. No. A9539-100G, Sigma-Aldrich) in DMEM medium). The spheroids were prepared as previously described[4] and the plates were incubated for 96 hours in a humidified atmosphere with 5% CO_2 in air at 37ºC. To generate spheroids with various sizes, single-cell suspensions form liquid overlay culture were seeded into agarose-coated plates with different cell numbers: 125, 250, 500, 1000, 1500, 2000, 3000 cells per well. The imaging of spheroids after 96 hours incubation was shown in **Supplementary Fig. 10**. Considering the penetration of cell membrane dye (CellMask™ Deep Red) and working distance of objective lens, we chose the spheroids with diameter of 90 to 110 µm (cell-seeding density: 125 cells per well) after 96 hours incubation to get the 3D imaging of whole spheroids and trajectories of EGFR tracking.

Fluorescence beads and EGFR. Both monolayer cells and spheroids were kept for additional 24 hours under serum-starvation condition before EGFR tracking. Plasma membrane was stained with CellMask™ and surface EGFRs were labeled with fluorescent nanoparticles (see schematic processes in **Supplementary Fig. 8**). In monolayer samples, cells was labeled using CellMask™ Deep Red Plasma membrane Stain (1:1000 dilution in DMEM; Cat. No. C10046, Life technologies) for 10 minutes at 37ºC. In spheroids, we carefully collected spheroids from 96-well plate using micropipettes and transferred them into 8-well chamber slides (8 to 10 spheroids per well). We stained spheroids with higher concentration of CellMask™ Deep Red (1:500 dilution) for 1 hour at 37 ºC because of the compact structure of spheroids. To label EGFRs with fluorescence, the cells were incubated with 1.5% BSA solution (1.5% bovine serum albumin, Cat. No. S7806, in PBS) for 15 minutes at 37ºC, and then EGFRs were recognized with biotin-labeled anti-EGFR antibodies (200 ng ml^{-1} in 1.5% BSA solution; Epidermal Growth Factor Receptor Ab-3, Cat. No. MS-311-B, Thermo Scientific) for 15 minutes at 37ºC. The antibody solution was removed and cells were washed twice using PBS. The stock solution of streptavidin-labeled ⌀40 nm fluorescent nanoparticles (FluoSpheres® Streptavidin-Labeled Microspheres, ⌀40 nm, red fluorescent, Cat. No. F8770, Life Technologies) was sonicated for 10 minutes and then diluted to 100 pM in DMEM. This solution was added into samples for 5 minutes at 37ºC, and the antibody-recognized EGFRs were visualized with the fluorescent nanoparticles. The samples were washed twice with PBS to remove unbound fluorescent nanoparticles. After labeling plasma membrane and EGFRs, we immediately put slides on our microscope and initiated the endocytosis of EGFR with DMEM containing 10 ng ml^{-1} EGF (recombinant human epidermal growth factor, Cat. No. PHG0311L, Life technologies). The internalization of fluorescent labeled EGFRs were observed after the stimulation of EGFs (**Supplementary Fig. 9**). Most of surface EGFRs were internalized into cytosol in 30 minutes after the stimulation of EGF[5], so we typically measured 2 to 4 trajectories from each well in 20 minutes and switched to another initiated sample. The volumes of all solutions and washing buffers used in staining were 200 µl per well. To inhibit endocytosis of EGFR (**Supplementary Fig. 15**), the spheroids were treated with 2 mM sodium azide (Cat. No. S8032, Sigma-Aldrich) in DMEM medium for 1 hour before the staining processes. Furthermore, cells with fluorescent labeled EGFRs were kept at 4ºC for 10 minutes before the stimulation of EGFs to inhibit EGFR internalization.

Data processing. All data processing was performed in Matlab (Mathworks, Natick). Raw data files of the trajectory are saved in binary format and contain positional information and count data for each time point. The data on every photon event can also be saved and resampled for higher time resolution, as explained in **Supplementary Fig. 11**. Each data point in the raw trajectory is a voltage output from the actuators (xy galvos and z stage) that must be converted to a physical distance. Conversion is performed by multiplying a gain factor for each axis. Trajectories are plotted by simply connecting lines between the points, no further post processing occurs.

2P LSM raw images are read into Matlab from binary files and denosied with a median filter before a 1D interpolation along the Z dimension. To segment the cellular compartments we use a simple intensity threshold technique that converts the image to a binary. Thresholds are selected at each z plane to account for variation in noise and brightness through the z-stack. The binary images are used as a mask to plot the cell isocontour. Because trajectories are measured with the same analog output device as the 2P LSM images they can be directly overlaid with the cell compartment isocontours with no conversion or scaling required.

Analysis of Diffusion Coefficient. The diffusion coefficients measured in **Supplementary Fig. 5** were calculated using the mean squared displacement for all time delays, $x(\tau)$, from the raw trajectory data,

$$6Dt = \langle (x(\tau) - x_0)^2 \rangle \ ,$$

Where D is the diffusion coefficient in $\mu m^2\ s^{-1}$, and t is the minimum time step. The diffusion coefficient parameter, D, was extracted from a linear regression of the MSD vs. τ curve.[6] The diffusion coefficients calculated from the experimental trajectory data were averaged over ~100 trajectories and the mean values were compared with the Stokes-Einstein equation for three dimensional diffusion of a spherical particle,

$$D = \frac{k_b T}{6\pi\eta r},$$

Where k_b is the Boltzmann constant, T is temperature, η is the dynamic viscosity, and r is the radius of the particle.

Supplementary References

1. Jones, S. a, Shim, S.-H., He, J. & Zhuang, X. Fast, three-dimensional super-resolution imaging of live cells. *Nat. Methods* **8,** 499–508 (2011).
2. Wells, N. P., Lessard, G. a & Werner, J. H. Confocal, three-dimensional tracking of individual quantum dots in high-background environments. *Anal. Chem.* **80,** 9830–4 (2008).
3. Durr, N. J. *et al.* Two-photon luminescence imaging of cancer cells using molecularly targeted gold nanorods. *Nano Lett.* **7,** 941–5 (2007).
4. Friedrich, J., Seidel, C., Ebner, R. & Kunz-Schughart, L. A. Spheroid-based drug screen: considerations and practical approach. *Nat. Protoc.* **4,** 309–24 (2009).
5. Sigismund, S. *et al.* Clathrin-mediated internalization is essential for sustained EGFR signaling but dispensable for degradation. *Dev. Cell* **15,** 209–19 (2008).
6. Di Rienzo, C., Gratton, E., Beltram, F. & Cardarelli, F. Fast spatiotemporal correlation spectroscopy to determine protein lateral diffusion laws in live cell membranes. *Proc Natl Acad Sci U S A* **110,** 12307–12312 (2013).
7. Thompson, M. a, Lew, M. D., Badieirostami, M. & Moerner, W. E. Localizing and tracking single nanoscale emitters in three dimensions with high spatiotemporal resolution using a double-helix point spread function. *Nano Lett.* **10,** 211–8 (2010).
8. Thompson, R. E., Larson, D. R. & Webb, W. W. Precise nanometer localization analysis for individual fluorescent probes. *Biophys. J.* **82,** 2775–83 (2002).
9. Warren, S. C. *et al.* Rapid Global Fitting of Large Fluorescence Lifetime Imaging Microscopy Datasets. *PLoS One* **8,** (2013).
10. Maus, M. *et al.* An experimental comparison of the maximum likelihood estimation and nonlinear least-squares fluorescence lifetime analysis of single molecules. *Anal. Chem.* **73,** 2078–86 (2001).
11. Patting, M., Wahl, M., Kapusta, P. & Erdmann, R. Dead-time effects in TCSPC data analysis. *Proc. SPIE* **6583,** 658307–658307–10 (2007).
12. Albota, M., Xu, C. & Webb, W. W. Two-Photon Fluorescence Excitation Cross Sections of Biomolecular Probes from 690 to 960 nm. *Appl. Opt.* **37,** 7352–7356 (1998).
13. Hess, S. T. & Webb, W. W. Focal Volume Optics and Experimental Artifacts in Confocal Fluorescence Correlation Spectroscopy. *Biophys. J.* **83,** 2003–2317 (2002).
14. Nagy, A., Wu, J. & Berland, K. M. Observation volumes and {gamma}-factors in two-photon fluorescence fluctuation spectroscopy. *Biophys. J.* **89,** 2077–2090 (2005).
15. Sahl, S. J., Leutenegger, M., Hilbert, M., Hell, S. W. & Eggeling, C. Fast molecular tracking maps nanoscale dynamics of plasma membrane lipids. *Proc. Natl. Acad. Sci.* **107,** 6829–6834 (2010).
